# Pathogenic variants of TUBB8 cause oocyte spindle defects by disrupting with EB1/CAKP5 interactions and potential treatment targeting microtubule acetylation through HDAC6 inhibition

**DOI:** 10.1002/ctm2.70193

**Published:** 2025-01-20

**Authors:** Hui Luo, Jianhua Chen, Cao Li, Tian Wu, Siyue Yin, Guangping Yang, Yipin Wang, Zhihan Guo, Saifei Hu, Yanni He, Yingnan Wang, Yao Chen, Youqiang Su, Congxiu Miao, Yun Qian, Ruizhi Feng

**Affiliations:** ^1^ State Key Laboratory of Reproductive Medicine and Offspring Health Nanjing Medical University Nanjing China; ^2^ Yangzhou Maternal and Child Health Care Hospital Affiliated to Yangzhou University Yangzhou China; ^3^ Clinical Center of Reproductive Medicine The Second Affiliated Hospital of Nanjing Medical University Nanjing China; ^4^ Shandong Provincial Key Laboratory of Animal Cells and Developmental Biology School of Life Sciences Shandong University Qingdao China; ^5^ Department of Reproductive Genetics Heping Hospital of Changzhi Medical College, Key Laboratory of Reproduction Engineer of Shanxi Health Committee Changzhi China; ^6^ Innovation Center of Suzhou Nanjing Medical University Nanjing Medical University Suzhou China

**Keywords:** D417N variant, female infertility, meiotic arrest, oocyte, TUBB8

## Abstract

**Background:**

Numerous pathogenic variants causing human oocyte maturation arrest have been reported on the primate‐specific TUBB8 gene. The main etiology is the dramatic reduction of tubulin α/β dimer, but still large numbers of variants remain unexplained.

**Methods:**

Using microinjection mRNA and genome engineering to reintroduce the conserved pathogenic missense variants into oocytes or in generating TUBB8 variant knock‐in mouse models, we investigated that the human deleterious variants alter microtubule nucleation and spindle assembly during meiosis. Live‐cell imaging and immunofluorescence were utilised to track the dynamic expression of microtubule plus end‐tracking proteins in vivo and analysed microtubule nucleation or spindle assembly in vitro, respectively. Immunoprecipitation‐mass spectrometry and ultramicro‐quantitative proteomics were performed to identify the differential abundance proteins and affected interactome of TUBB8 protein.

**Results:**

First, we observed a significant depletion of the EB1 signal upon microinjection of mutated TUBB8 mRNA (including R262Q, M300I, and D417N missense variants), indicating disruption of microtubule nucleation caused by these introduced TUBB8 missense variants. Mechanically, we demonstrated that the in vivo TUBB8‐D417N missense variant diminished the affinity of EB1 and microtubules. It also harmed the interaction between microtubules and CKAP5/TACC3, which are crucial for initiating microtubule nucleation. Attenuated Ran‐GTP pathway was also found in TUBB8‐D417N oocytes, leading to disrupted spindle assembly. Stable microtubule was largely abolished on the spindle of TUBB8‐D417N oocytes, reflected by reduced tubulin acetylation and accumulated HDAC6. More importantly, selective inhibition of HDAC6 by culturing TUBB8‐D417N oocytes with Tubacin or Tubastatin A showed morphologically normal spindle and drastically recovered polar‐body extrusion rate. These rescue results shed light on the strategy to treat meiotic defects in a certain group of TUBB8 mutated patients.

**Conclusion:**

Our study provides a comprehensive mechanism elucidating how TUBB8 missense variants cause oocyte maturation arrest and offers new therapeutic avenues for treating female infertility in the clinic.

## Graphical abstract

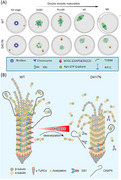

## 1 INTRODUCTION

Infertility affects approximately 17.5% of the adult population in their reproductive age.^1^, ^2^ Contributing to about half of the incidence, female infertility impacts over 40 million women worldwide.^3^ Assisted reproductive technology (ART) has enabled numerous infertile couples to successfully conceive. However, recurrent ART failures due to oocyte maturation arrest or fertilisation failure pose significant challenges for large numbers of patients, bringing substantial economic and psychological burdens. Therefore, unravelling the molecular basis of oocyte‐defect‐based infertility is crucial for developing therapeutic strategies and advancing precision medicine. Through whole‐exome sequencing technology and human genetic analysis, causal relationships were established between monogenic missense variants and female infertility. In 2016, TUBB8 was revealed to be responsible for oocyte metaphase I (MI) arrest.^4^ Subsequently, pathogenic genes including PATL2, TLE6, and WEE2 were identified as contributors to oocyte maturation arrest, fertilisation failure, and embryonic arrest, respectively.^5^, ^6^, ^7^ These findings highlight the critical role of genetic factors in this disease. Currently, infertility genetic screening (IFGS) utilising next‐generation sequencing has become an integral component of diagnosing affected infertile patients. The results obtained from genetic diagnosis serve as an essential foundation for clinical diagnosis and treatment.

Missense variants in TUBB8 are estimated to account for approximately 30% of oocyte meiosis I arrest patients.^8^ These missense variants impair microtubule dynamics and disrupt the proper assembly of the oocyte meiotic spindle, leading to profound oocyte maturation arrest and female infertility. Microtubules are essential cytoskeletal components in all eukaryotes and comprise α/β‐tubulin heterodimer subunits that assemble into hollow tubular filaments and structures.^9^ Although the microtubule structure is highly conserved in eukaryotes, α/β‐tubulin exists in different isotypes, with humans having nine α‐tubulin and ten β‐tubulin subtypes.^10^, ^11^ The primate‐specific *TUBB8* gene was found to be exclusively expressed in oocytes and early embryos, and encoded the most abundantly expressed β‐tubulin isotype in these cells. Recent studies have expanded the known TUBB8 mutational spectrum associated with female infertility, screening hundreds of pathogenic variants.^12^, ^13^, ^14^, ^15^, ^16^, ^17^ Earlier evidence showed that some missense variants affect chaperone‐dependent folding and α/β‐tubulin heterodimer assembly, leading to a dramatic decrease of α/β‐tubulin dimer.^4^ However, for the rest of the missense variants that did not reduce the α/β‐tubulin dimer production, the reason for their defective oocyte spindle assembly with seemingly sufficient yields of α/β‐tubulin dimers is not yet known. Furthermore, therapeutic strategies for most of the missense variants remain ineffective due to the dominant‐negative nature of these pathogenic missense variants. Altogether, a deeper and more comprehensive understanding of TUBB8 missense variants causing oocyte maturation arrest and female infertility is urgently needed.

In eukaryotes, microtubule spindle orchestrated chromosome alignment and segregation during cell division. In mitotic cells, canonical centrosomes consist of a pair of centrioles surrounded by pericentriolar material. They are the main microtubule organising centres in centrosomal spindles.^18^ These structures function as the primary microtubule‐organising centres within the centrosomal spindles. They play a crucial role as the main loci for microtubule nucleation and constitute the two poles of the mitotic spindles. Unlike somatic cells, mammalian oocytes segregate chromosomes with a specialised microtubule spindle that lacks centrosomes.^19^ Instead, oocytes have evolved mechanisms to nucleate microtubules independently of centrosomes, enabling the establishment of a bipolar spindle in both human and rodent mammals. At the onset of meiotic resumption, mouse oocytes contain multiple acentriolar MTOCs (aMTOCs) serve as a major site of microtubule nucleation, and functionally replace centrosomes.^20^ These aMTOCs consist of partial centrosomal proteins and microtubule nucleation factors, containing γ‐tubulin, TACC3, and CKAP5. Upon nuclear envelope breakdown, multiple aMTOCs are fragmented into a large number of small aMTOCs and GTP‐bound RAN promotes an extensive increase in microtubule nucleation from aMTOCs.^20^, ^21^ Subsequently, the self‐assembly of microtubules into a bipolar spindle occurs through the action of plus end‐directed motor proteins with distributed aMTOCs at both poles of the bipolar spindle.^21^ Precise regulation of microtubule nucleation and proper spindle assembly is crucial for ensuring oocyte nuclear maturation, as any abnormalities in these processes can lead to oocyte meiotic arrest.^4^, ^22^ Therefore, we propose that a special fraction of TUBB8 missense variants, which do not impair α/β‐tubulin dimer production, disrupt oocyte maturation arrest and result in female infertility.

In the present study, we aim to better understand the functional diversification of this oocyte maturation pathogenic gene TUBB8. We explored how spindle assembly was disorganised by TUBB8 missense variants that barely affect α/β‐tubulin heterodimer yields and why meiotic arrest occurs in mutant oocytes. To this end, we analysed the process of microtubule nucleation in different TUBB8 missense variants that can cause oocyte meiotic arrest. These missense variants caused severe microtubule nucleation disruption and abnormal spindle assembly. With an in vivo oocyte‐specific TUBB8‐D417N missense variant knock‐in mouse model, we propose that mutant TUBB8 tubulin heterodimers undergo intrinsic alterations that interfere with the interaction between critical microtubule‐associated proteins and microtubules, and ultimately result in microtubule instability and dysregulated microtubule dynamics. As an initial attempt at potential pharmaceutical treatment, we also tested and validated the rescue effects of small‐molecule inhibitors of tubulin acetylation, which could be promising therapeutic targets for oocyte defects caused by genetic factors. Our study provided a tremendous advance in understanding and treating female infertility associated with TUBB8 missense variants.

## 2 MATERIALS AND METHODS

### 2.1 Ribo‐RNA‐lite data analysis of human oocytes and embryos

Transcriptome (RNA‐Seq) and translatome (Ribo‐Seq) data from human oocytes and early embryos were downloaded from previous study,^23^ encompassing germinal vesicle (GV) oocytes, metaphase I (MI) oocytes, metaphase II (MII) oocytes, 1‐cell (1C), 2‐cell (2C), 4‐cell (4C) and 8‐cell embryos, as well as inner cell mass (ICM) from the blastocysts and human embryonic stem cells (hESC). The translational efficiency (TE) of each gene was defined as the following formula:

$$\mathrm{TE}\;=\log_{2}\frac{FPKM_{Ribo-Seq}}{FPKM_{RNA-Seq}}.$$

### 2.2 Mice

All animal procedures were approved by the Institutional Animal Care and Use Committee of Nanjing Medical University and were conducted following the institutional guidelines for the Animal Care and Use Committee of Nanjing Medical University. CRISPR/Cas9‐based genome engineering was employed to construct C‐terminal 3×HA‐tagged TUBB8‐WT^flox/flox^ and TUBB8‐D417N^flox/flox^ knock‐in mouse strains (C57BL/6J). Gdf9‐Cre mice were obtained from Youqiang Su Laboratory (Shandong University) and maintained on identical C57BL/6J genetic backgrounds. To generate oocyte‐specific TUBB8‐WT and TUBB8‐D417N knock‐in mice, female mice carrying the TUBB8‐WT^flox/flox^ (hereafter referred to as WT) and TUBB8‐D417N^flox/flox^ (hereafter referred to as D417N) alleles were crossed with Gdf9‐Cre males. Genotyping for LoxP and Cre was carried out using PCR amplification. Mice were genotyped by PCR using primers for TUBB8 (WT or D417N) LoxP (Forward: 5′‐CTGGATCACAGG TGTGGAGT‐3′, Reverse: 5′‐GGCTACATGCAGGCAAAC‐3′), and Gdf9‐Cre (Forward: 5′‐ TCTGATGAAGTCAGGAAGAACC‐3′, Reverse: 5′‐ GAGATGTCCTTCACTCTGATTC‐3′). These mice were accommodated in an environment featuring a 12‐h light‐dark cycle with consistent temperature conditions, where they had unlimited access to food and water. The maintenance and utilisation of all animals adhered strictly to the ethical guidelines outlined by the Nanjing Medical University's Institutional Animal Care and Use Committee, with the ethical approval number IACUC‐1912016.

### 2.3 Preparation and culture of mouse oocytes

All animal experiments performed in this study were approved by the Institutional Animal Care and Use Committee (IACUC) of Nanjing Medical University. GV oocytes were obtained from the ovaries of 3‐ to 5‐week‐old females at 46 to 48 h post‐injection with 7.5 IU pregnant mare serum gonadotropin (PMSG, Ningbo Second Hormone Factory, China). GV oocytes were collected from antral follicles by puncturing them with a fine needle and subsequently cultured in M16 medium (Sigma‐Aldrich, M7292). To prevent meiotic resumption during collection, the M16 medium supplemented with 200 µM 3‐Isobutyl‐1‐methylxanthine (Sigma‐Aldrich, IBMX, I7018) was covered with paraffin oil (Sigma‐Aldrich, M8410) and maintained at a temperature of 37°C. Oocytes were matured for 7 h for metaphase I and 15 h for metaphase II experiments after oocytes were released into IBMX‐free M16 medium at 37°C. Oocytes were collected after culture for about 2, 3 and 4 h, corresponding to stage of at GVBD, MTOC recruitment and pro‐metaphase I, respectively. Upon release into IBMX‐free M16 medium at 37°C, oocytes were matured for 7 h for metaphase I and 15 h for metaphase II.

### 2.4 Expression constructs, in vitro transcription of RNAs, and microinjection

The plasmids encoding H2B‐mCherry^24^ and EB3‐EGFP^25^ plasmids were synthesised by the MiaoLing Plasmid Platform. The wild‐type TUBB8 expression plasmids, carrying the FLAG tag and designated as pCMV6‐TUBB8‐FLAG, were kindly donated by Professors Lei Wang and Qing Sang of Fudan University. Mutations in TUBB8 were introduced utilising the Mut Express II Fast Mutagenesis Kit (Vazyme, C214). Linearised plasmids were transcribed in vitro using the HiScribe™ T7 ARCA mRNA Kit (NEB, E2060S) to generate mRNAs, following the manufacturers' protocol. Synthesised RNAs were purified with Monarch® RNA Cleanup Kits (NEB, T2040S) and stored at –80°C until needed for further experimentation.

Mouse GV oocytes were microinjected with RNAs as previously described.^4^ H2B‐mCherry mRNA was a needle concentration (final concentration in the microinjection needle) of 50 ng/µL, EB3‐EGFP mRNA at 300 ng/µL, TUBB8‐WT and missense variants mRNAs at 200 ng/µL. After microinjection, oocytes were allowed to express mRNA for 20 h before being released into the IBMX‐free M16 medium.

### 2.5 Cell lysis, immunoprecipitation, and immunoblotting

HeLa cells were cotransfected C‐terminal AviTag WT or D417N of TUBB8 pCMV6 vectors (pCMV6‐TUBB8‐AviTag) and pEF1a‐BirA‐V5‐neo, following the previously described protocol.^16^ Detailed cell lysis and immunoprecipitation procedures were conducted according to previous studies.^16^ Briefly, to prepare whole‐cell extracts for immunoprecipitation, PBS‐washed cells were incubated on ice with a lysis buffer containing 20 mM Tris‐HCI, 150 mM KCL, 0.2% Triton X‐100, 1 mM EGTA, 4 mM MgCI2, 10% Glycerine, and 4% Cocktail. After centrifugation, the supernatant was incubated with pre‐blocked Dynabeads™ M280 Streptavidin (Invitrogen, 11205D) beads at 4°C for 2 h. The beads were then washed seven times with wash buffer (20 mM Tris‐HCL, 150 mM KCL, 0.2% Triton X‐100, 1 mM EGTA, 4 mM MgCI_2_, 10% Glycerine) and denatured by boiling for 5 min in 1× SDS loading buffer. The co‐immunoprecipitated proteins were eluted by 1× SDS sample and analysed by immunoblotting as indicated.

### 2.6 Live‐cell time‐lapse imaging

Oocytes were allowed to express mRNAs for a duration of 8 h prior to their release into IBMX‐free M2 medium for the purpose of imaging. For live imaging, the GV oocytes expressing H2B‐mCherry and EB3‐EGFP mRNA were recorded under a Nikon Ti2‐E‐C2+ confocal microscope (Nikon, Japan) equipped with an environmental chamber set to 37°C and infused with 5% O_2_, 5% CO_2_ and 90% N_2_. Time points were acquired at 15‐min intervals. Subsequently, images were exported and analysed using the image processing software associated with the Andor spinning disk confocal microscope.

### 2.7 Immunofluorescence and confocal microscopy

Oocytes were fixed, permeabilised, and blocked for routine immunofluorescence. In brief, oocytes were fixed in 2% paraformaldehyde overnight and then permeabilised in phosphate‐buffered saline (PBS) with 0.5% triton X‐100 (PBT) for 20 min at room temperature. After blocking in PBS with 3% BSA (PBS‐BSA) for 2 h at RT, oocytes were incubated with primary antibodies. Alternatively, for immunostaining of CREST (Human anti‐Centromere, Antibodies Incorporated, 15–234) and NuMA (Abcam, ab97585), oocytes were fixed and permeabilised with cold methanol for 5 min at –20°C. For cold‐stable microtubule assay, oocytes were kept on ice for 6 min before fixation. All antibody incubations were performed in PBS‐BSA at 5 mg/mL overnight at 4°C (for primary antibodies) and at 20 mg/mL for 2 h at RT (for secondary antibodies). Primary antibodies used were mouse anti‐EB1 (Biosciences, 610534), rabbit anti‐CKAP5 (Proteintech, 26457‐1‐AP), rabbit anti‐DCTN1 (Proteintech, 55182‐1‐AP), mouse anti‐Pericentrin (BD Biosciences, 611814), rabbit anti‐HA (Abcam, ab9110), mouse anti‐HA (MBL, M180‐3), rabbit anti‐TACC3 (Abcam, ab51976), rabbit anti‐TPX2 (Proteintech, 11741‐1‐AP), rabbit anti‐RanGAP1 (Abcam, ab92360), rabbit anti‐γ‐Tubulin (Sigma‐Aldrich, T6557), mouse anti‐active Ran (Ran‐GTP) (Neweast Bioscience, 26915), rabbit anti‐Ran (Proteintech, 10469‐1‐AP), Cy3‐β‐Tubulin (Sigma‐Aldrich, C4585), rabbit anti‐α‐Tubulin (Alexa Fluor® 488 Conjugate) (CST, 5063S), mouse anti‐α‐Tubulin (Sigma‐Aldrich, T9026), rabbit anti‐HDAC6 (Immunoway, YT2118), rabbit anti‐αTAT1 (Proteintech, 28828‐1‐AP), rabbit anti‐alpha Tubulin (acetyl K40) (Abcam, ab179484), mouse anti‐alpha Tubulin (acetyl K40) (Alexa Fluor® 647 Conjugate) (Abcam, ab218591), rabbit anti‐Acetylated Tubulin (Sigma‐Aldrich, T7451), mouse anti‐Detyrosinated Tubulin (Sigma‐Aldrich, AB3201), rat anti‐Tyrosinated Tubulin (Sigma‐Aldrich, MAB1864), mouse anti‐BubR1 (BD Biosciences, 612502), rabbit anti‐KIF11 (Sigma‐Aldrich, HPA010568), mouse anti‐FLAG‐Cy3 (Sigma‐Aldrich, A9594); Secondary antibodies used were Alexa Fluor 488‐conjugated Highly Cross‐Adsorbed anti‐rabbit (Invitrogen, A11034), Alexa Fluor 594‐conjugated Highly Cross‐Adsorbed anti‐mouse (Invitrogen, A11032), Alexa Fluor 647 conjugated Highly Cross‐Adsorbed anti‐rabbit (Abcam, ab150083), Cy3‐labelled Goat Anti‐Rat IgG (H+L) (Beyotime, A0507), and Cy3 AffiniPure Fab Fragment Donkey anti‐Human (H+L) (Jackson Immunoresearch, 709‐166‐149). DNA was stained with DAPI (Beyotime, C1005).

Wild‐type and missense variants of TUBB8, tagged with a C‐terminal FLAG epitope, were expressed in HeLa cells through transfection using Lipofectamine 2000 (Invitrogen, 1168019), following the manufacturer's instructions. After 48 h, the cells were collected and fixed for immunofluorescence analysis as described.

Oocytes and HeLa cells were mounted on glass slides for confocal imaging using an SP8 laser scanning microscope (Leica). Immunofluorescence intensity was measured by acquiring images of control and experimental groups under identical imaging parameters on the same microscope, followed by data analysis using Image J software. Confocal images were acquired as z‐stacks at 0.5 µm intervals to ensure visualisation of the entire meiotic spindle and are presented as sum intensity z‐projections to show the spindle.

### 2.8 Immunoblotting

Immunoblotting analysis was performed according to previously described methods.^16^ Briefly, the protein sample was denatured by boiling at 95°C for 5 min. The protein lysates were separated on SDS‐PAGE gels and transferred onto polyvinylidene difluoride (PVDF) membranes for subsequent probing of the target proteins. The membrane was blocked with 5% BSA for 1 h at room temperature, followed by incubation with primary antibodies diluted in 5% BSA (in 1× TBST). Finally, chemiluminescence using ECL was employed for image acquisition. The primary antibodies used included mouse anti‐EB1 (Biosciences, 610534), rabbit anti‐CKAP5 (Proteintech, 26457‐1‐AP), HRP‐Streptavidin (Beyotime, A0303), rabbit anti‐HDAC6 (Immunoway, YT2118), mouse anti‐GAPDH (Abcam, ab8245), and rabbit anti‐alpha Tubulin (acetyl K40) (Abcam, ab179484), rabbit anti‐CKAP5 (Proteintech, 26457‐1‐AP), rabbit anti‐TPX2 (Proteintech, 11741‐1‐AP), rabbit anti‐RanGAP1 (Abcam, ab92360), mouse anti‐active Ran (Ran‐GTP) (Neweast Bioscience, 26915), rabbit anti‐Ran (Proteintech, 10469‐1‐AP). Secondary antibodies used were HRP‐conjugated goat anti‐mouse IgG (YIFEIXUE BIOTECHNOLOGY, YFSA01) or HRP‐conjugated mouse anti‐rabbit IgG (Sangon Biotech, D110065).

### 2.9 In vitro translation assays

In in vitro translation assays, plasmids specifically designed for T7‐driven expression of both wild‐type and D417N variant of TUBB8 were utilised to facilitate expression with the TnT ® T7 Quick Coupled Transcription/Translation System (L1170, Promega), supplemented with biotinylated‐labelled tRNA according to the manufacturer's protocol. At designated time points, a 1 µL aliquots of the translation reaction were mixed with protein sample buffer and analysed by Native‐PAGE. For the final lane depicted on both gels, the reactions were supplemented with native bovine brain tubulin at a final concentration of 1 µM after 90 min, followed by an additional incubation period of 60 min to promote the formation of native tubulin heterodimers. For non‐denaturing PAGE, the reactions were loaded and analysed using native Invitrogen NativePAGE 3 to 12% Mini Protein Gels (BN1001BOX, Invitrogen), and then transferred onto PVDF membrane. Following blocking in 5% BSA in TBS plus 0.1% Tween, membranes were then incubated overnight at 4°C with HRP‐Streptavidin (Beyotime, A0303) in 5% BSA TBST then washed 3 × 10‐min TBST and acquired images using Sparkjade ECL super (Sparkjade) reagent.

### 2.10 Protein purification and mass spectrometry

At 42 h post‐transfection, KGN cells that ectopically expressed the C‐terminal AviTag‐fused wild‐type (WT) or D417N mutant of TUBB8 encoded by pCMV6 vectors and pEF1a‐BirA‐V5‐neo were harvested. The protocols for cell harvest and protein purification were performed as indicated above. Subsequently, label‐free mass spectrometry was conducted by BioTeke Corporation to facilitate the analysis of the proteins.

### 2.11 Proteomic analysis

For low‐input single‐cell proteome profiling, oocytes were first stripped of their zona pellucida using warm acidic Tyrode solution and then thoroughly washed three times with DPBS. One hundred WT and D417N oocytes were lysed in 5 µL lysis buffer containing 8 M urea and 1% EDTA‐free protease inhibitor. The protein solution underwent reduction with 5 mM dithiothreitol for a duration of 30 min at 56°C, followed by alkylation with 11 mM iodoacetamide in the dark at room temperature for 15 min. Subsequently, the protein sample was diluted by incorporating 100 mM TEAB until the urea concentration fell below 2 M. Digestion was initiated by adding trypsin at a mass ratio of 1:50 (trypsin‐to‐protein) and allowed to proceed overnight. This was followed by a second digestion with a trypsin‐to‐protein mass ratio of 1:100 for an additional 4 h. Ultimately, the peptides obtained were desalted using a C18 SPE column.

The tryptic peptides were dissolved in solvent A, consisting of 0.1% formic acid and 2% acetonitrile in water, and subsequently loaded onto a custom‐made reversed‐phase analytical column (25‐cm length, 75/100 µm i.d.). Separation of the peptides was achieved using a gradient, which began at 6% solvent B (acetonitrile with 0.1% formic acid) and increased to 24% over 70 min, then to 35% in the next 14 min, and finally reached 80% within 3 min, maintaining this concentration for the last 3 min. Following this, the peptides underwent capillary‐based separation and were then subjected to mass spectrometry analysis using the timsTOF Pro instrument (Bruker Daltonics).

High‐resolution TOF was utilised for the detection and analysis of peptide precursor ions and their subsequent fragments, with a scanning range of 100–1700 *m*/*z*. Data acquisition was conducted in parallel accumulation serial fragmentation (PASEF) mode. Following the acquisition of the primary mass spectrum, three rounds of PASEF scans were employed to gather the secondary spectrum, focusing on precursor ions with charge states ranging from 0 to 5.

Precursors and fragments were analysed at the TOF detector, with MS/MS scan range from 100 to 1700 *m*/*z*. The timsTOF Pro was operated in parallel accumulation serial fragmentation (PASEF) mode. Precursors with charge states 0 to 5 were selected for fragmentation, and 10 PASEFMS/MS scans were acquired per cycle. The dynamic exclusion was set to 30 s. A dynamic exclusion duration of 30 s was imposed during tandem mass spectrometry to prevent redundant scanning of the precursor ions.

The MS/MS data underwent processing via the MaxQuant search engine (version 1.6.15.0). The human SwissProt database (consisting of 20 422 entries) served as the basis for tandem mass spectrometry searches. Initially, a mass tolerance of 20 ppm was applied to the precursor ions, which was then tightened to 5 ppm in the subsequent main search, with a fragment ion tolerance of 0.02 Da. Carbamoylmethylation on Cys was designated as a fixed modification, while acetylation at the protein's N‐terminus and oxidation of Met were considered variable modifications. The false discovery rate (FDR) was adjusted to below 1%.

For bioinformatics analysis, differentially abundant proteins (fold change greater than 3/2 and less than 2/3) were enriched based on GO and KOG databases using a two‐tailed Fisher's exact test for all identified proteins.

### 2.12 Enrichment network analysis

Due to the huge number of GO ontologies, to find the key ontology, simplifyEnrichment (v.1.12.0)^26^ and aPEAR (v.1.0.0)^27^ packages were used to cluster the GO terms.

### 2.13 Hub networks and hub genes analysis

The STRING database (https://string‐db.org/) was utilised to upload the target proteins for constructing a protein‐protein interaction (PPI) network. This network was then visualised and analysed using Cytoscape software (version 3.9.0).^28^ The top 30 hub genes within the PPI network were further abstracted by CytoHubba plugin^29^ using MCC algorithms. The node size represents the –log_2_Ratio value. A larger node indicates a greater downregulation change since all proteins in the plot are downregulated.

### 2.14 Human and mouse homolog gene analysis

The human and mouse homologous genes were switched by biomaRt package^30^ (v.2.58.0) to analyse the conservation and divergence of differentially abundant proteins between human and mouse. The alluvial plot of the homologous gene correspondence between the two species was drawn by the ggalluvial package (v.0.12.5).^31^

### 2.15 Model generation and structure analysis

For illustrative purposes, a high‐resolution cryo‐EM structure of the microtubule with kinesin KIF11 (PDB ID: 6TA4) was used to demonstrate the binding or conformational changes of β‐Tubulin to KIF11 after D417N mutation. In addition, to visualise the effects of altered residues on protein structure, we obtained the structure prediction of the TUBB8 with kinesin KIF11 from AlphaFold2. These structures were visualised by Pymol.

### 2.16 Focused ion beam–scanning electron microscopy (FIB‐SEM) and transmission electron microscopy (TEM)

For transmission electron microscopy staining, oocytes were fixed at 2.5% EM‐grade glutaraldehyde at 4°C for 2 h. Subsequently, the oocytes were embedded in 1.5% agarose (Amresco, N605), and the agarose gel cube (1 × 1 × 1 mm) containing oocytes was fixed overnight in 2.5% EM‐grade glutaraldehyde. Following post‐fixation with 1% osmium tetroxide for 1 h, the samples were rinsed with 0.1 m PBS three times, for 15 min each time. After being replaced with pure acetone twice for 15 min, the samples underwent dehydration with gradient acetone of 50%, 70%, 90%, and 100%, each step lasting for 15 min. Subsequently, oocytes were processed and embedded in epoxypropane resin according to standard TEM procedures. Ultrathin sections of 70 nm thickness were performed and subjected to staining through the uranyl acetate‐lead citrate double dyeing approach. Finally, oocyte samples were acquired and images acquisition by focused ion beam‐scanning electron microscopy (Helios G4 CX) and transmission electron microscopy (JEM‐1400Flash).

### 2.17 Measurement of hormones

Blood samples were obtained via retro‐orbital bleeding. The sera were then isolated through centrifugation of the clotted blood at 4600 rpm for 15 min, as previously described.^32^ The levels of follicle‐stimulating hormone (FSH) and luteinising hormone (LH) were measured using radioimmunoassay by the Beijing North Institute of Biotechnology (XH‐6080 radio‐immunoanalyzer).

### 2.18 Inhibitor treatment

All inhibitors were dissolved in DMSO (Sigma‐Aldrich, D2650) as 1000× stocks. To selectively inhibit HDAC6, mouse GV oocytes were cultured in an M16 medium supplemented with Tubacin (MCE, HY‐13428) at a concentration of 2 µM or Tubastatin A (MCE, HY‐13271A) at a concentration of 10 µM. To specifically inhibit HDAC8, GV oocytes were cultured in an M16 medium containing PCI‐34051 (MCE, HY‐15224) at a concentration of 10 µM. Taxol (MCE, HY‐B0015) was added to the culture medium at a final concentration of 10 µM to induce polymerisation of spindle microtubules. Metaphase I oocytes were exposed to 10 µM nocodazole (HY‐13520) for 1 h to depolymerise the microtubule.

### 2.19 Microtubule depolymerise and repolymerise

For microtubule regrowth, oocytes initially underwent the 60‐min nocodazole treatment to depolymerise microtubules, followed by a wash into a drug‐free medium. Subsequently, oocytes were incubated for durations of 30, 60, and 90 min, then fixed and immunostained for further analysis.

### 2.20 Expression of TUBB8 in HeLa cells

The wild‐type and mutant forms of TUBB8, tagged with a C‐terminal FLAG epitope, were expressed in HeLa cells through transfection using Lipofectamine 2000 (Invitrogen, 1168019) following the manufacturer's instructions. After 48 h of transfection, the cells were collected, fixed, permeabilised, and subjected to immunofluorescence staining using antibodies against FLAG (Cy3‐conjugated) (Sigma‐Aldrich, A9594), α‐Tubulin (CST, 5063S), and acetylated α‐Tubulin (K40) (647‐conjugated) (Abcam, ab218591). Microtubule phenotypes were quantified by examining 200 cells expressing either wild‐type or mutant TUBB8 in each of the three independent experiments.

### 2.21 Analysis of microtubule post‐translational modifications

The modification data of α‐tubulin and β‐tubulin from human and mouse were downloaded from The Tubulin Database (https://tubulindb.bio.uci.edu/). The Tubulin Database extracts a reference catalogue of multispecies tubulin post‐translational modifications from public datasets, primary literature and predictive algorithms.^33^ We summarised the total number of various modifications in different subtypes of α‐tubulin and β‐tubulin. The results were exhibited by bubble maps through ggplot2 package (v.3.4.4).^34^

### 2.22 Foldseek for predicting TUBB8 orthologs

The protein structures for human TUBB8 were initially predicted using ColabFold. Subsequently, Foldseek was employed to perform a one‐to‐all structure‐based search, matching the predicted structures against entries in the AlphaFold Database (AFDB) and the Protein Data Bank (PDB).

### 2.23 Microtubule depolymerise and IP‐MS

To identify the key spindle regulator mediated by TUBB8, we employed transient co‐transfection of TUBB8‐WT‐AviTag and BirA in HEK293T cells. To synchronise mitosis for investigating microtubules and microtubule‐associated proteins for IP‐MS, the transfected HEK293T cells were treated with a very low concentration of nocodazole (100 ng/mL) before harvesting.^35^, ^36^, ^37^ Subsequently, microtubules were depolymerised through cold treatment for 10 min, followed by immunoprecipitation using M280‐Dynbeads and subsequently analysed through mass spectrometry.

The tryptic peptides were dissolved in 0.1% formic acid (solvent A) and subjected to analysis using a Nano LC system (EASY‐nLC 1200, Thermo Scientific), which was interfaced with a QE‐HFX mass spectrometer. Separation of the peptides was achieved on an analytical column (Acclaim PepMap® RSLC, C18; 75 µm × 15 cm, 3 µm particle size, 100 Å pore size; Thermo Scientific) through a linear gradient of 3–35% buffer B (consisting of 80% acetonitrile with 0.1% formic acid) at a flow rate of 0.3 µL/min over 65 min. The mass spectrometer was operated in data‐dependent analysis (DDA) mode with a dynamic exclusion duration of 30 s. MS spectra were acquired within the *m*/*z* range of 350–1500 at a resolution of 60 000 at *m*/*z* 200. Subsequently, the most intense ions were fragmented within a cycle time of 1 s using high‐energy collisional dissociation (HCD) with a normalised collision energy (NCE) of 30.0. MS/MS scans were acquired at a resolution of 15 000 at *m*/*z* 200. The resulting raw data were imported into Proteome Discoverer (Version 2.4, Thermo Scientific) for detailed protein identification.

### 2.24 Statistical analysis

All experiments were independently conducted at least three times, and the data are presented as mean ± SEM via Prism software (GraphPad Software). Statistical analysis of differences between the two groups was performed using Student's *t*‐test. One‐way ANOVA with Šidák correction for multiple comparisons, with *p* values denoted as *p < .05, **p < .01, ***p < .001, and ****p < .0001 to indicate significance levels. Nonsignificant results are indicated as ns.

## 3 RESULTS

### 3.1 Defective microtubule polymerisation caused by compromised binding of varied TUBB8 missense variants to key factors in mouse oocytes

**FIGURE 1:**
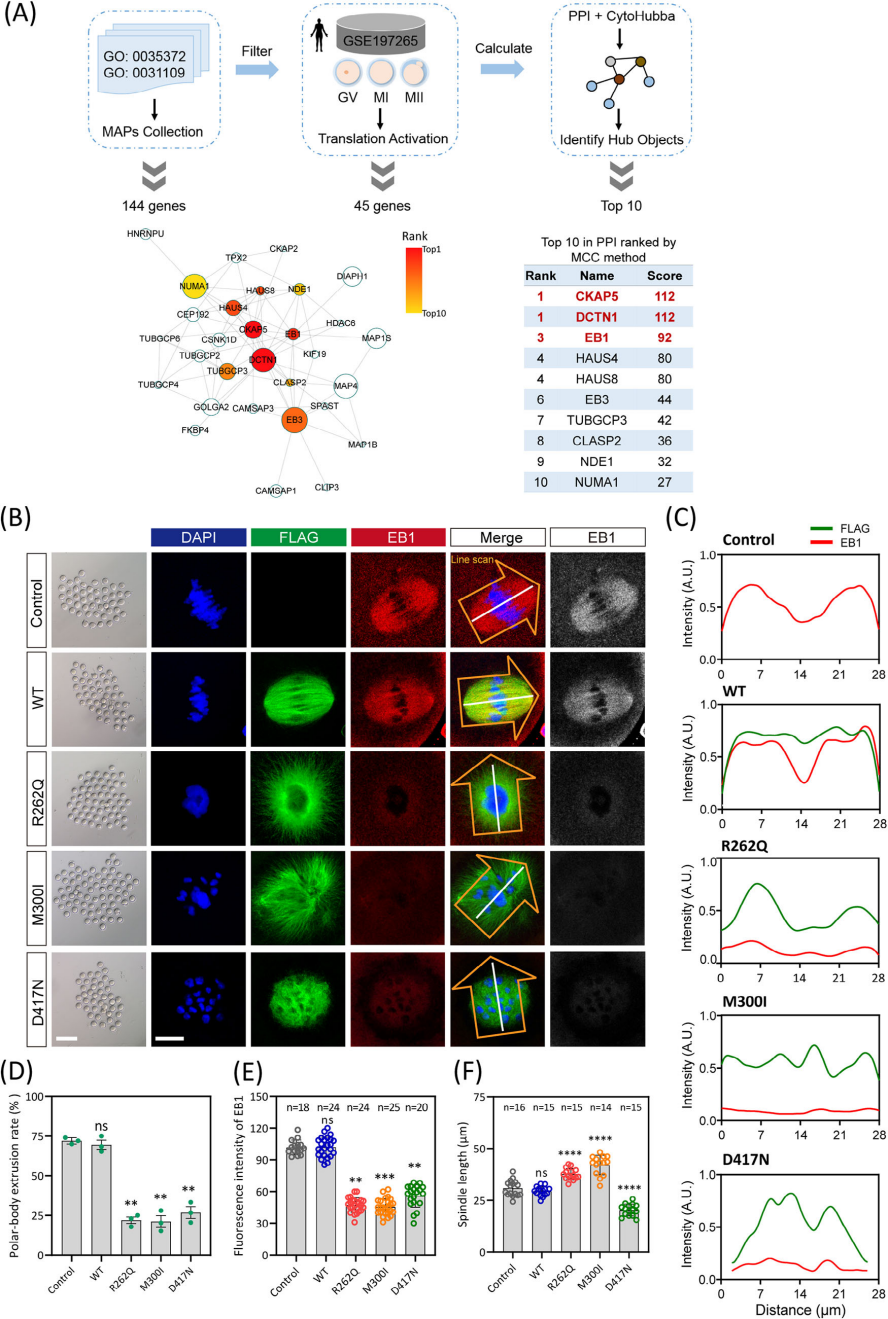
Impaired EB1 binding observed in mouse oocytes injecting pathogenic mutated TUBB8. (A) Flow chart of functional screening of key factors involved in oocyte microtubule dynamics. (B) Light microscopy and representative immunofluorescence images of mouse oocytes microinjected with Flag‐tagged RNA encoding wild‐type (WT) or mutant forms of TUBB8 are shown. Control indicates mouse GV oocytes without injection. GV oocytes were injected with TUBB8‐WT mRNA (200 ng/µL) (*n* = 80), or missense variant mRNAs (200 ng/µL) R262Q (*n* = 96), M300I (*n* = 105), D417N (*n* = 72), and maintained in IBMX for 20 h before being washed into IBMX‐free medium to allow meiotic resumption. Metaphase I oocytes were then immunostained with DAPI to visualise chromosomes (blue), FLAG to visualise TUBB8 expression (green), and EB1 labelling growing microtubules (red). Scale bars in light microscopy images and immunofluorescence images represent 200 µm and 10 µm, respectively. (C) Intensity profiles of TUBB8‐FLAG and EB1 along the white line in panel A are depicted. (D) Polar‐body extrusion rates were quantitatively analysed in mouse oocytes injected with mutated RNA compared to WT control, represented as mean ± SEM values. (E) Quantitative analysis of fluorescence intensity of EB1 is presented as the mean ± SEM. Numbers indicate the individual oocytes quantified. (F) Statistical analysis of spindle length in mouse oocytes expressing different TUBB8 mutations. Numbers indicate the individual oocytes quantified. *p*‐values were calculated using One‐way ANOVA with Šidák correction for multiple comparisons to panels C, D, and E (**denotes *p* < .01, *** indicates *p* < .001, **** indicates *p* < .0001).

Previous studies have shown that the main disease‐causing mechanism of TUBB8 missense variants is the dramatic reduction of tubulin α/β dimer, whereas there is still a great fraction of missense variants that remain elusive.^4^, ^38^ Hereby, we focused on those TUBB8 missense variants with comparable α/β‐tubulin dimers to wild‐type and compromised oocyte spindle assembly, targeting two crucial events governing oocyte maturation: microtubule nucleation and spindle assembly, and where these events are primarily regulated by microtubule dynamics. To identify the key factors reasonable for microtubule dynamics, we extracted 144 microtubule‐associated proteins (MAPs) that fulfilled the Gene Ontology (GO) term ‘microtubule polymerisation or depolymerisation (GO: 0031109)’ and ‘protein location to microtubule (GO: 0035372)’ (Figure 1A). Considering the GV oocytes showed transcript repression but became actively translated upon meiotic resumption, in which genes regulated nuclear and cytoplasmic maturation underwent highly dynamics.^23^ Thus, we used translatome data during oocyte maturation to filter the translation‐activated MAPs and identified 45 translation‐activated genes (Figure 1A). Subsequently, based on protein‐protein interaction analysis and the MCC algorithm, we recognised the top ten hub factors controlling microtubule polymerisation (Figure 1A). Among them, CKAP5, DCTN1, and EB1 were the top three candidates (Figure 1A). We then investigated their function for microtubule nucleation using oocytes expressing TUBB8 pathogenic missense variants.

We injected flag‐tagged wild‐type and mutated TUBB8 mRNA into mouse oocytes and examined the expression of EB1, the well‐known stabiliser at the plus‐ends of microtubules during polymerisation. Consistent with earlier research, oocytes injected with wild‐type TUBB8 mRNA exhibited normal meiotic maturation with a bipolar spindle (Figure 1B). In contrast, injection of mutated TUBB8 mRNA (R262Q, M300I, and D417N) led to significant depletion of the EB1 signal (Figure 1C–E). This was accompanied by disorganised or defective spindle formation and a notable decrease in the polar body extrusion rate compared to the wild‐type group (Figure 1B and D). In addition, compared with the spindle of oocytes expressing wild‐type TUBB8, the spindle of oocytes expressing missense variants exhibited more disordered morphology, showing longer or shorter spindle length (Figure 1F). We also analysed the expression of a specialised plus‐end tracking protein DCTN1 in oocytes with WT or TUBB8 missense variants. We found that its expression pattern was consistent with that of EB1, which was significantly lower than that of wild‐type TUBB8, and had no obvious localisation at the spindle poles (Figure S1A and C). Furthermore, we evaluated the expression of a microtubule nucleation factor CKAP5 and found that the expression levels of the two missense variants (R262Q and M300I) were significantly reduced. In contrast, the D417N variant did not exhibit a significant change in expression (Figure S1B and D). These findings indicated that introducing these TUBB8 missense variants in mouse oocytes was responsible for the disruption of microtubule nucleation.

### 3.2 Disrupted EB1 binding in TUBB8‐D417N knock‐in mouse oocytes with maturation arrest

**FIGURE 2:**
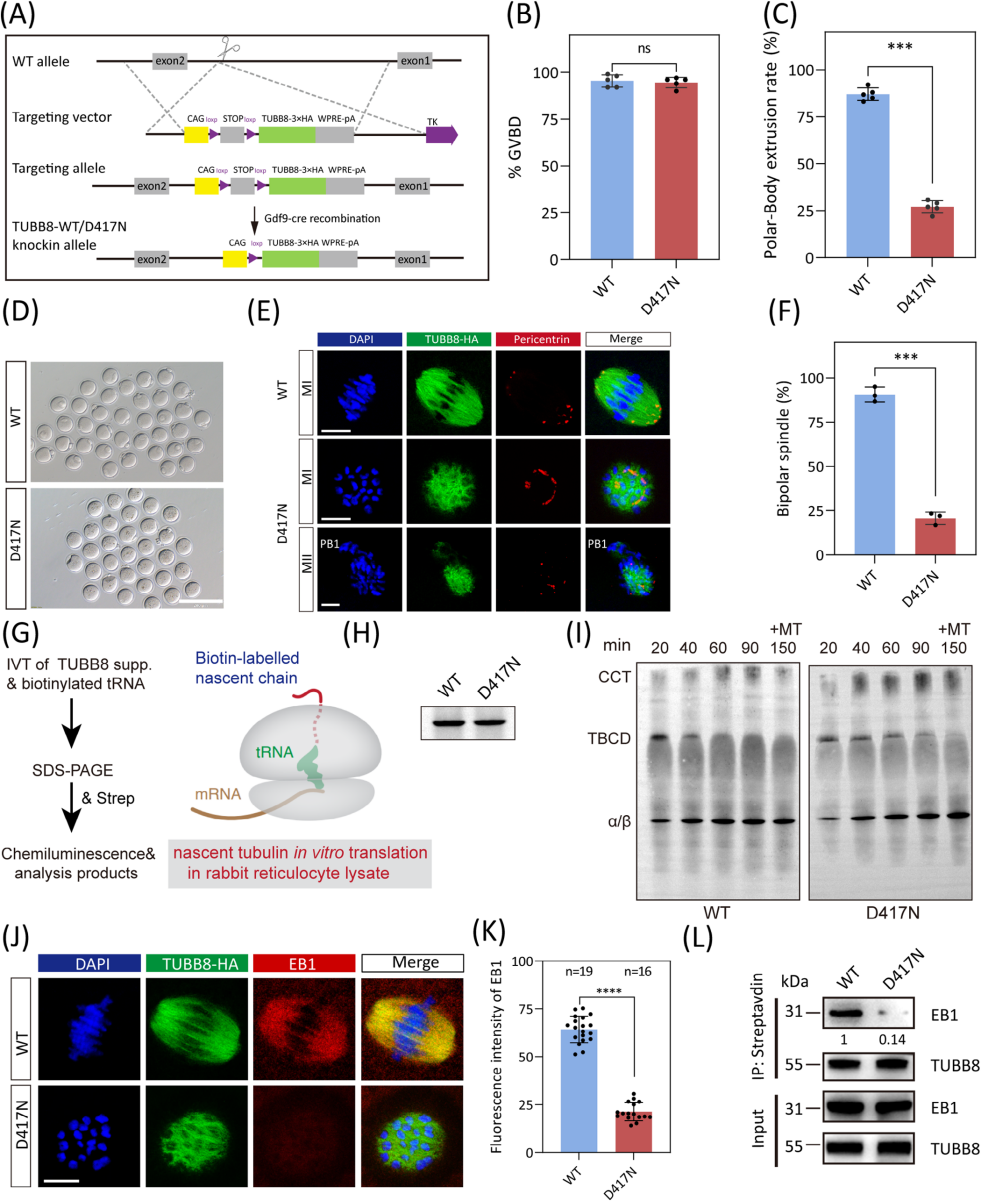
Impaired EB1 binding with TUBB8‐D417N causes mouse oocyte maturation arrest in vivo without α/β tubulin dimer yield change. (A) Schematic diagram showing the strategy of TUBB8‐WT and TUBB8‐D417N conditional knockin. (B) Quantification of the percentage of oocytes that undergo nuclear envelope breakdown (GVBD) in vitro (Unpaired Student's t test, two‐tailed, ns = no significance). Graphs show the mean ± SEM from five independent experiments. (C) Quantification of the percentage of oocytes that undergo PBE in vitro (Unpaired Student's *t*‐test, two‐tailed, ***p < .0001). (D) Representative light microscopy images showing oocytes from WT and D417N females. Scale bar, 200 µm. (E) Representative immunofluorescence images demonstrating spindle assembly in mouse metaphase I or metaphase II oocytes from WT and D417N females. Green, microtubule (TUBB8‐HA); red, aMTOCs (Pericentrin); blue, DNA (DAPI). Scale bar, 10 µm. (F) Quantification of the percentage of oocytes that assembled bipolar spindle (Unpaired Student's *t*‐test, two‐tailed, ***p < .001). (G) Experimental strategy to detect α/β tubulin heterodimer assembly products produced by in vitro translation in rabbit reticulocyte lysate. (H) Native gel of in vitro translated reaction products for WT and D417N mutant. (I) A kinetic analysis presented the tubulin folding on non‐denaturing gels derived from wild‐type and D417N variant translation products. (J) Representative immunofluorescence images of mouse metaphase I oocytes. Green, microtubule (TUBB8‐HA); red, EB1; blue, DNA (DAPI). Scale bar, 10 µm. (K) Quantitative analysis of mean EB1 fluorescence intensity for WT and D417N oocytes. Data were presented as the mean  ±  SEM. Numbers indicate the individual oocytes quantified. ***p   < .001 by unpaired Student's *t*‐test. (L) Co‐immunoprecipitation was performed to determine the decreased affinity between TUBB8 and EB1 by D417N missense variant. The immunoblots of protein precipitants were probed with EB1 and streptavidin (TUBB8) antibodies.

A previous comprehensive study showed that some missense variants affect chaperone‐dependent folding and α/β‐tubulin heterodimer assembly, leading to decreased α/β‐tubulin dimer levels and abnormal spindle formation (e.g. R262Q and M300I).^4^ However, other variants like D417N do not reduce α/β‐tubulin dimer production, and their impact on oocyte spindle assembly remains unclear.^4^ Intriguingly, the D417N variant occurs in a conserved site critical for motor binding within the kinesin superfamily and has been linked to multiple diseases across different tubulin isotypes. Furthermore, previous findings revealed that the introduce of the D417N pathogenic variant into mouse oocytes recapitulated the unique spindle phenotype observed in human eggs.^4^ Therefore, we generated oocyte‐specific expressed wild‐type TUBB8‐WT ^flox/flox^; Gdf9‐Cre (hereafter referred to as WT) and TUBB8‐D417N ^flox/flox^; Gdf9‐Cre (hereafter referred to as D417N) mice (Figures 2A and S1E). The conditional knock‐in of TUBB8 did not affect the normal physiological functions of mice, as demonstrated by their comparable development, body weight, number of ovulated oocytes, serum level of FSH, and serum level of LH to those of the C57BL/6 group (Figure S1F–J). The G to A transition at nucleotide 1251 in exon 4 caused the pathogenic D417N missense variant. While the germinal‐vesicle breakdown (GVBD) rate was not affected in the TUBB8‐D417N mice, there was a significant reduction in the polar‐body extraction rate compared to WT (Figure 2B–D). Unlike WT oocytes that mature normally and stay in metaphase II with bipolar spindles, about 75% of D417N oocytes are arrested at the metaphase I stage accompanied by an abnormal spindle (Figure 2E and F). Consistent with previous observations that this missense variant does not disrupt tubulin heterodimer assembly (Figure S1H), but may exert downstream dominant effects on microtubule dynamics. As expected, the recruitment and distribution of pericentrin, a classic marker of aMTOCs, were weakened and disrupted, indicating severely impacted microtubule nucleation by carrying the D417N missense variant (Figure 2E and F).

The de novo assembly of tubulin heterodimers depends on the concerted action of multiple chaperones, including five exclusive factors for this pathway. To gain insight into the molecular mechanisms by which the D417N variant disrupts tubulin function, we investigated the chaperone‐dependent α/β‐heterodimer assembly products produced by in vitro translation in rabbit reticulocyte lysate (Figure 2G). Our data revealed no differences in translational efficiency in vitro, the kinetics of folding, or the yield of α/β‐heterodimer formation compared to the wild type (Figure 2H and I). Thus, these data imply that this missense variant does not disrupt tubulin heterodimer assembly but may exert downstream dominant effects on microtubule dynamics, such as microtubule nucleation and spindle assembly.

The precise correlation between microtubule growth and nucleation suggested that the stabilisation of microtubule dynamics was facilitated by the presence of plus‐end factors. Therefore, we performed immunofluorescence analysis to evaluate in vivo expression levels of EB1. Our findings demonstrated a significant decrease in EB1 expression within D417N oocytes compared to WT, consistent with our previous observations from in vitro microinjection (Figure 2J and K). In addition, co‐immunoprecipitation assays investigating the TUBB8‐EB1 interaction demonstrated a sharp decline in their affinity caused by the D417N missense variant (Figure 2L). These results confirm that TUBB8‐D417N causes mouse oocyte spindle defects and meiosis I arrest in vivo. This finding aligns with the phenotype of human oocyte maturation arrest seen in patients with infertility carrying the p.D417N missense variant.^4^

To investigate the impact of the D417N variant on oocyte meiosis, we employed live‐cell imaging to evaluate the progression of meiosis and the dynamics of spindle assembly in both WT and D417N oocytes. By co‐injecting mRNA encoding fluorescently fused histone H2B (mCherry) and the plus‐end marker EB3 (EGFP), which serves as a reliable indicator of microtubule growth in live cells, we were able to monitor microtubule growth and nucleation. In WT oocytes, time‐lapse imaging revealed normal microtubule nucleation, spindle assembly, and chromosome segregation (Figure S2A). However, the presence of the D417N missense variant led to a significant decrease in EB3 signal and resulted in metaphase I arrest in oocytes due to severe disruption of microtubule nucleation and spindle assembly (Figure S2B). Furthermore, a loose distribution of chromosomes was observed in D417N oocytes, indicating impaired microtubule‐dependent chromosome behavior (Figure S2B). These results confirm that meiotic arrest occurs due to microtubule nucleation within D417N oocytes, resulting in severe impacts during spindle assembly at metaphase I.

### 3.3 A combination of regulators contributed to TUBB8 D417N‐mutant oocytes with defective microtubule nucleation

Previous study has shown that CKAP5 (also known as ch‐TOG) is a crucial cofactor of EB1, binding to EB1 and promoting microtubule polymerisation.^39^ We conducted immunofluorescence analysis to investigate the localisation and expression of CKAP5 during meiosis. We found that CKAP5 is a marker for aMTOCs, colocalising with the established aMTOC component Pericentrin (Figure S3A). Before GVBD, CKAP5 is located in the oocyte cortex and then moves toward the nuclear membrane before it ruptures (Figure S3A). Following GVBD, CKAP5 is recruited to initiate spindle microtubule nucleation and fragments into smaller aMTOCs, evenly distributed on opposite sides of bipolar spindle poles (Figure S3A and B). Both unfragmented and stretched aMTOCs colocalise with microtubules extending along the nuclear envelope (Figure S3B).

**FIGURE 3:**
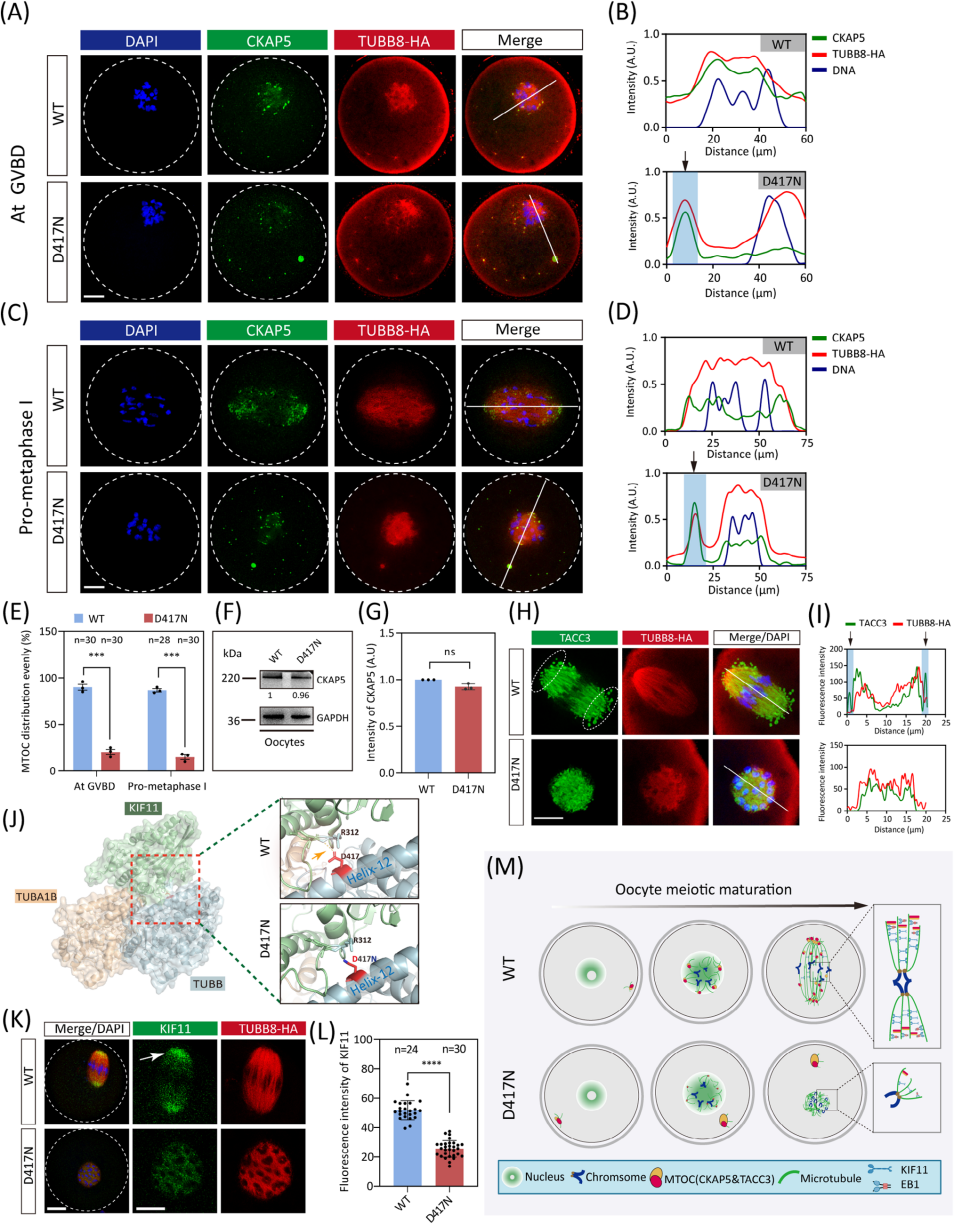
Impaired microtubule nucleation caused by dysregulation of key factors in TUBB8 D417N carrying oocytes. (A to D) Representative immunofluorescence images of mouse oocytes at different stages of meiosis. Representative images at GVBD and pro‐metaphase I are shown in (A) and (C) respectively. Green, CKAP5; red, microtubule (TUBB8‐HA); blue, DNA (DAPI). Intensity profiles along the white lines are shown in (B), and (D) for oocytes at GVBD and pro‐metaphase I, respectively. The dashed line demarcates the oocyte. Black arrows indicate that CKAP5 is peripheral positioning in D417N oocytes, whereas it is perinuclear distribution in WT oocytes. Scale bars, 10 µm. (E) Quantification of the percentage of oocytes with the perinuclear distribution of CKAP5 at GVBD stage and pro‐metaphase I. Graphs show the mean ± SEM from three independent experiments (Unpaired Student's t test, two‐tailed, ns = no significance, ***p < .001). (F) Western blot analysis of protein levels of CKAP5 in WT and D417N oocytes. The blots were probed with CKAP5 and GAPDH antibodies. Each sample had 50 oocytes. (G) Quantitative analysis of the mean intensity of CKAP5 (Unpaired Student's *t*‐test, ns = no significance). (H) Representative immunofluorescence images of the TACC3 localisation in mouse oocytes at metaphase I. Green, TACC3; red, microtubule (TUBB8‐HA); blue, DNA (DAPI). The dashed line denotes the LISD structure. Scale bar, 10 µm. (I) Intensity profiles of TACC3 along the white lines are shown in (H). Black arrows indicate the LISD distribution which only presents TACC3 signals. (J) Structural implications of amino acid substitutions in KIF11 with a/β‐Tubulin. The structure of kinesin protein KIF11 and α/β‐tubulin heterodimers is depicted. Mutant residue in β‐tubulin are highlighted in blue. Yellow arrow indicates the salt bridge between β‐Tubulin and KIF11. (K) Representative immunofluorescence images of the KIF11 localisation in mouse oocytes at metaphase I. Green, KIF11; red, microtubule (TUBB8‐HA); blue, DNA (DAPI). Scale bar, 10 µm. (L) Quantitative analysis of mean KIF11 fluorescence intensity for WT and D417N oocytes. Data were presented as the mean  ±  SEM. Numbers indicate the individual oocytes quantified. ***p   < .001 by Unpaired Student's *t*‐test. (M) The mechanism model of D417N missense mutation leading to abnormal microtubule nucleation. The microtubule instability is due to a D417N missense mutation that prevents the stability of stable EB1 binding, thereby disrupting the dynamic interaction between the plus end of the microtubule and the nucleating CKAP5, which is necessary to initiate microtubule nucleation and spindle assembly.

CKAP5 (aMTOC) is perinuclear before meiotic division so that it can be readily distributed around the chromatin to initiate microtubule nucleation upon GVBD (Figure S3B). While CKAP5 undergoes fragmentation at GVBD in WT oocytes, D417N oocytes exhibit an aberrant peripheral positioning of CKAP5 that persists outside the nuclear periphery (Figure 3A). The distribution profiles demonstrated unevenly abnormal CKAP5 in D417N (Figure 3B). Subsequently, perinuclear CKAP5 fragmented into multiple small aMTOCs at GVBD before reclustering at the two spindle poles in WT oocytes (Figure 3C). In contrast, the aberrant distribution persists throughout pro‐metaphase I and metaphase I, leading to meiosis I arrest in D417N oocytes (Figures 3C and D and S3C). Quantitative analysis revealed significantly impaired perinuclear distribution of CKAP5 in D417N oocytes compared to WT oocytes at the GVBD and pro‐metaphase I stages (Figure 3E). The D417N missense variant did not alter CKAP5 expression in D417N oocytes or the interaction between TUBB8 and CKAP5 (Figures 3F and G and S3D), indicating that the CKAP5 function of binding free tubulin heterodimers remained unaffected.

Recent research has demonstrated that TACC3, combined with CKAP5 and several key factors, actively participates in microtubule nucleation within human oocytes.^22^ Therefore, we postulated that the expression of TACC3 would also abnormally localise in D417N missense variant oocytes. To rule out this hypothesis, we visualised TACC3 and observed a similar expression pattern and localisation to CKAP5 (Figure S4A). As expected, similar peripheral accumulation was observed for TACC3 in D417N oocytes, indicating impaired microtubule nucleation and spindle assembly caused by the D417N missense variant (Figure S4B). TACC3 is a crucial component of the liquid‐like meiotic spindle domain (LISD) within oocyte spindles and is essential for efficient microtubule assembly to form stable acentrosomal spindles.^40^ In WT oocytes, intense signals of TACC3 were detected along the microtubules as well as prominent LISD protrusions at both spindle poles; however, no LISD protrusions were observed at any poles in D417N mutant oocytes, implying impaired microtubule nucleation and spindle assembly caused by the D417N missense variant (Figure 3H and I).

Since kinesin proteins drive aMTOC fragmentation and spindle assembly,^21^, ^41^ we further investigated whether aberrant aMTOC resulted from decreased activity of KIF11 (a key kinesin protein promoting aMTOC fragmentation and spindle bipolarisation) in D417N oocytes. Indeed, structural evidence suggests that the D417N missense variant would severely impact the binding between β‐tubulin and KIF11 (Figure 3J). To investigate the potential impact of the TUBB8 missense variant, AlphaFold2 was utilised to predict the interaction structure between TUBB8 and KIF11, and we found the pathogenic D417N variant does seriously affect the binding of KIF11 to TUBB8 (Figure S4C). Furthermore, our findings indicate that the D417N missense variant leads to a reduction in KIF11 signalling at the spindle poles, resulting in the formation of malformed spindles in D417N oocytes due to decreased kinesin protein affinity (Figure 3K and L). It has been demonstrated that suppression of KIF11 causes a significant decrease in bivalent tension.^42^ Consistent with this, we observed reduced stretching of bivalents in oocytes with the D417N missense variant, as demonstrated by measuring the distance between pairs of sister kinetochores from each bivalent (Figure S4D and E). These results suggest that the D417N missense variant affects the binding or recruitment of KIF11 to microtubules, leading to inadequate stretching of the bivalents.

To directly demonstrate that the D417N mutation disrupts microtubule nucleation, we depolymerised spindle microtubules acutely with nocodazole. Our results showed that in the control group, microtubules gradually regrowth after nocodazole removal and rapidly reassembled into well‐structured bipolar spindles. In contrast, oocytes with the D417N mutant could only form morphologically disrupted spindles (Figure S5). This finding suggested that the D417N mutation have a significant dampening impact on normal microtubule growth and repolymerisation in an α/β‐Tubulin heterodimer assembly‐independent manner.

Next, we planned to identify in oocytes whether key regulators were binding to TUBB8 microtubule lattice or depolymerised tubulins. (Figure S6A and B). Extracts of HEK293T cells expressing TUBB8‐WT with a biotinylation tag (AviTag) together with the biotin ligase BirA were used for streptavidin pull‐down assays. Subsequently, microtubules were depolymerised through cold treatment, followed by immunoprecipitation and mass spectrometry (IP‐MS) analysis to identify regulatory factors binding to microtubule rather than depolymerised tubulins in the control group without cold treatment. Differentially abundant proteins were identified with a parameter of log2 FoldChange | > 1.5 and padj  <  0.05. We found that 625 out of 1344 detected proteins in the cold treatment group showed significantly different abundance compared with their levels in the control group, among them 17 were more and 608 were less in the cold treatment group (Figure S6B). Among them, several tubulin proteins were identified as binding partners of TUBB8, including one γ‐tubulin (TUBG1), three α‐tubulin isotypes (TUBA1C, TUBA3D, TUBA8), and four β‐tubulin isotypes (TUBB, TUBB4B, TUBB3, TUBB6) (Figure S6C–F). It is noteworthy that the protein abundances of TUBG1, TUBA8 and TUBB6 significantly plunged after cold treatment, indicating the representative tightly bound γ‐tubulin, α‐tubulin and β‐tubulin isotypes to TUBB8 microtubule lattice, respectively. By performing gene ontology (GO) pathway enrichment analysis with these 608 decreased proteins, we found that proteins involved in mitochondrial protein‐containing complex and ATP hydrolysis activity, as well as ribosome‐related proteins, were dramatically less bind to TUBB8 after cold treatment, indicating that these organelles may gather around the spindle during cell division in preparation for cell division and organelle recombination (Figure S6G–I).

Strikingly, there was a marked downregulation of several microtubule regulatory factors, including the previously detected CKAP5, KIF11, and DCTN1 in comparison to the control group, indicating more binding was found to TUBB8 microtubules than tubulins (Figure S6J–L). We then validated in mouse TUBB8‐WT knock‐in oocytes the key microtubule‐spindle regulatory factors CKAP5, KIF11, and DCTN1 through immunofluorescence staining after cold treatment and Nocodazole treatment (Figure S6J–L). These results showed that the expression and location of these key regulatory factors disappeared along with the disruption of microtubules after both treatments, indicating tighter binding was found to TUBB8 microtubules lattice than tubulins.

Collectively, these findings suggest that the destabilisation of microtubules occurs due to the prevention of the stability of EB1 stable binding by the D417N missense variant, which disrupts dynamic interaction between microtubule plus ends and nucleator CKAP5 necessary for initiating microtubule nucleation and spindle assembly (Figure 3M).

### 3.4 Perturbed Ran‐GTP pathway in TUBB8‐D417N oocytes resulted in spindle assembly

**FIGURE 4:**
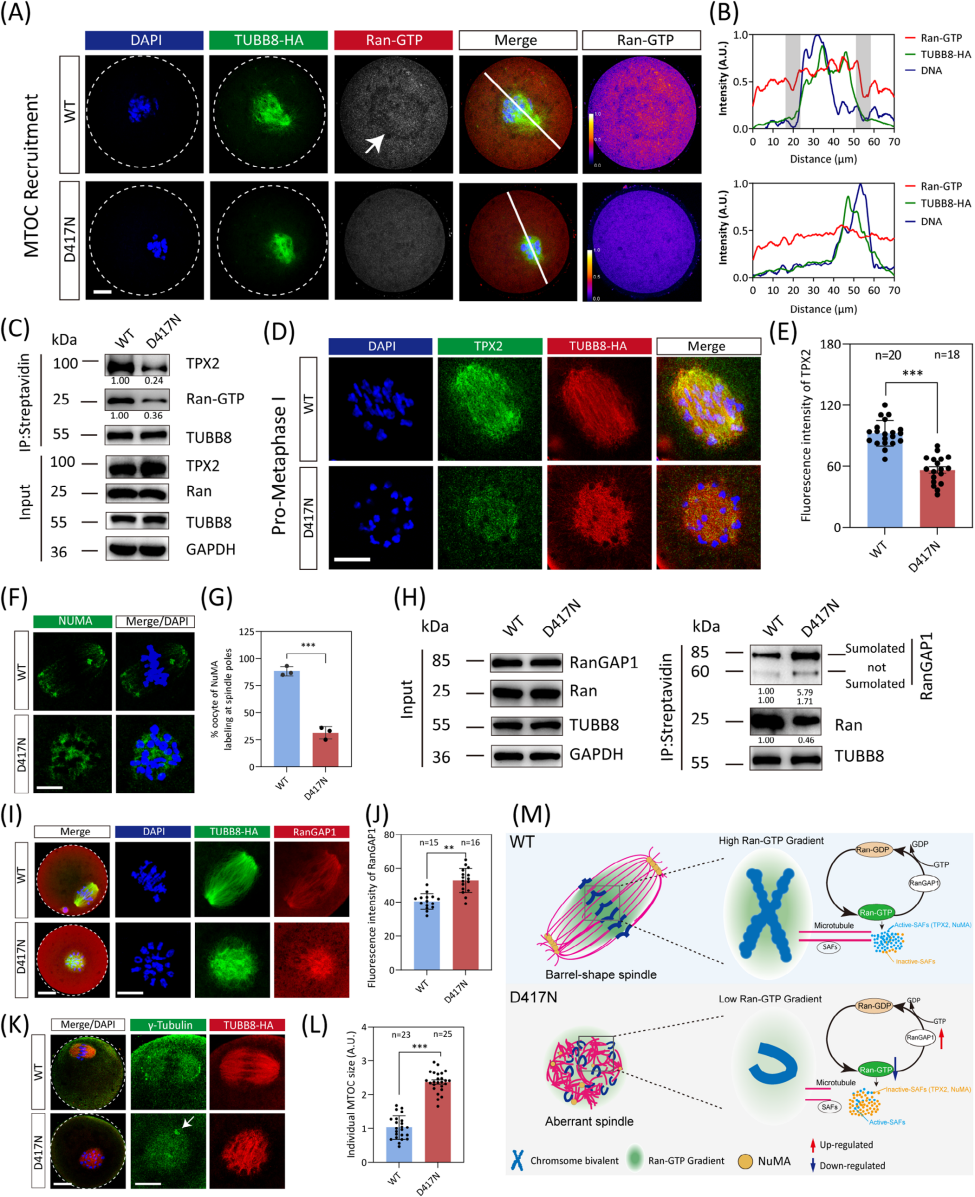
Defective spindle assembly caused by perturbed Ran‐GTP pathway in TUBB8 D417N oocytes. (A) Representative immunofluorescence images of mouse oocytes at MTOC recruitment stages of meiosis. Green, microtubule (TUBB8‐HA); red, active‐Ran (Ran‐GTP); blue, DNA (DAPI). The dashed line demarcates the oocyte. Right panels showing LUT colour grading according to intensity of the Ran‐GTP. (B) Intensity profiles along the white lines are shown for oocytes at the MTOC recruitment stage. The shaded box represents the Ran‐GTP gradient in WT oocyte. (C) Co‐immunoprecipitation was performed to determine the decreased affinity between TUBB8 and Ran‐GTP or TPX2 by the D417N missense variant. The immunoblots of protein precipitants were probed with Ran‐GTP, TPX2, and streptavidin (TUBB8) antibodies. (D) Representative immunofluorescence images of mouse oocytes at the Pro‐MI stage. Green, TPX2; red, microtubule (TUBB8‐HA); blue, DNA (DAPI). Scale bars, 10 µm. (E) Quantitative analysis of the mean intensity of TPX2 (***p   < .001 by Unpaired Student's *t*‐test). Numbers indicate the individual oocytes quantified. (F) Representative immunofluorescence images of mouse oocytes at MI stage. Green, NuMA; blue, DNA (DAPI). Scale bars, 10 µm. (G) Quantitative analysis of the percentage of oocytes with NuMA labelling at spindle poles (***p   < .001 by unpaired Student's *t*‐test). (H) Co‐immunoprecipitation was performed to determine the increased affinity between TUBB8 and RanGAP1 by the D417N missense variant. The immunoblots of protein precipitants were probed with RanGAP1 and streptavidin (TUBB8) antibodies. (I) Representative immunofluorescence images demonstrating spindle assembly in mouse metaphase I oocytes from WT and D417N. Green, microtubule (TUBB8‐HA); red, RanGAP1; blue, DNA (DAPI). Scale bar, 10 µm. (J) Quantitative analysis of the mean intensity of RanGAP1 (***p   < .001 by unpaired Student's *t*‐test). Numbers indicate the individual oocytes. (K) Representative immunofluorescence images demonstrating spindle assembly in mouse metaphase I oocytes from WT and D417N females. Green, γ‐Tubulin; red, microtubule (TUBB8‐HA); blue, DNA (DAPI). Scale bar, 10 µm. The white arrow indicates the unfragmented MTOC. (L) Quantification of individual MTOC size (Unpaired Student's *t*‐test, two‐tailed, *** *p* < .001). Numbers indicate the individual oocytes quantified. (M) The mechanism model of the D417N missense mutation disrupted spindle assembly. During spindle assembly, Ran‐GTP recruits spindle assembly factors by creating a concentration gradient around the chromosomes. The D417N missense mutation led to a reduction in active Ran‐GTP and an abnormal increase in RanGAP1, causing the imbalance between Ran‐GTP and Ran‐GDP. This further resulted in the abnormal release of spindle assembly factors TPX2 and NuMA, leading to assemble the spindle ineffectively.

Subsequently, we aimed to investigate the impact of the D417N missense variant on spindle assembly. Previous research has demonstrated the essential role of Ran‐GTP in spindle assembly in both mouse and human oocytes.^43^ Therefore, we evaluated the Ran‐GTP expression pattern in oocytes from WT and D417N females. While a distinct ring‐shape of active Ran localised around chromosomes after GVBD was clearly observed in WT oocytes, this ring was severely disrupted and the overall Ran‐GTP expression was largely decreased by the presence of the D417N missense variant (Figures 4A and B and S7). Furthermore, co‐immunoprecipitation assays using HeLa cells revealed that compared to WT, the D417N missense variant exhibited reduced affinity for Ran‐GTP (active‐Ran) (Figure 4C). It has been proposed that high concentrations of Ran‐GTP surrounding chromatin led to the local release of spindle assembly factors (SAFs) such as TPX2 and NuMA, activating them to nucleate microtubules and assemble spindles.^44^, ^45^, ^46^ Consistent with low expressions of Ran‐GTP, TPX2 in D417N mutant oocytes was significantly decreased and led to the failure of NuMA to localise at the spindle poles (Figure 4D–G).

Given that RanGAP1 drives the gradient of Ran‐GTP in mitosis by converting Ran‐GTP to Ran‐GDP, we speculate whether the imbalance between Ran‐GTP and Ran‐GDP is caused by the over‐activity of RanGAP1. Our observation showed that the D417N missense variant significantly increased the conversion of Ran‐GTP to Ran‐GDP by RanGAP1 compared to WT (Figure 4H–J). To determine the crucial role played by the D417N missense variant in spindle assembly, we subsequently evaluated spindle assembly through immunofluorescence staining analysis on fixed oocytes. When WT oocytes undergo maturation to metaphase I, the spindles elongate while the chromosomes align at the metaphase I plate and multiple aMTOCs fuse together to form two well‐defined poles (Figure 4K). However, D417N mutant oocytes exhibit a persistent microtubule ball with unresolved chromosomes (Figure 4K). Additionally, we observed larger γ‐tubulin foci (aMTOCs), indicating a failure in fragmenting aMTOCs (Figure 4K and L). These findings suggest that the D417N missense variant dramatically disrupts spindle assembly through the Ran‐GTP pathway (Figure 4M).

### 3.5 Altered interactome and decreased microtubule acetylation in TUBB8 D417N variant

**FIGURE 5:**
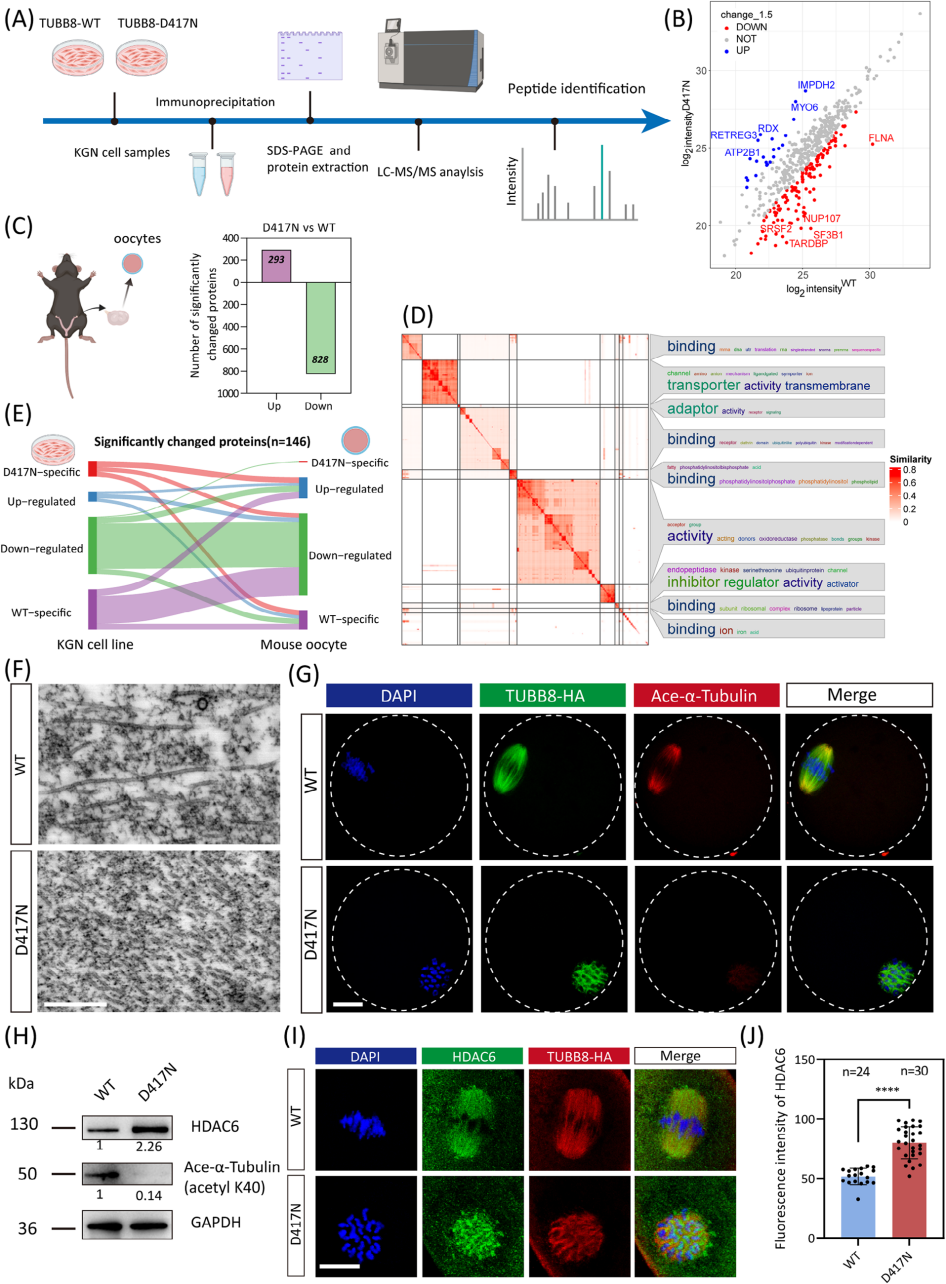
TUBB8‐D417N missense variant leads to altered interactome and decreased microtubule acetylation levels and abnormal spindle assembly. (A) Workflow to identify the significantly changed proteins by D417N missense variant through quantitative proteomic analysis. (B) The volcano map shows a significantly altered protein between WT and D417N missense variant. (C) The bar graph indicates the number of differentially abundant proteins between WT and D417N oocytes. (D) The similarity heatmap illustrates word clouds that clustered and annotated from 934 GO terms enriched in downregulated proteins. (E) Alluvial diagram showing 4 groups of genes with corresponding genes expressed in human KGN cell line and mouse oocyte homologous genes. (F) Focused ion beam–scanning electron microscopy (FIB‐SEM) displayed that the microtubules in the missense variant extended were short and microtubule breakage. Scale bars, 400 nm. (G) Representative immunofluorescence images of mouse metaphase I oocytes. Green, microtubule (TUBB8‐HA); red, acetylated microtubule (ace‐α‐Tubulin); blue, DNA (DAPI). (H) Western blot analysis of protein levels of ace‐α‐Tubulin and HDAC6 in WT and D417N oocytes. The immunoblots of protein precipitants were probed with HDAC6, ace‐α‐Tubulin (K40), and GAPDH antibodies. Each sample contained 50 oocytes, with GAPDH serving as a loading control. (I) Representative immunofluorescence images of mouse metaphase I oocytes. Green, HDAC6; red, microtubule (TUBB8); blue, DNA (DAPI). (J) Quantitative analysis demonstrates the mean fluorescence intensity of HDAC6 from panel (I) (Unpaired Student's *t*‐test, two‐tailed, *** *p* < .0001). Numbers indicate the individual oocytes quantified.

To further investigate the effect of the interactome of the TUBB8 protein, we performed quantitative proteomic analyses on immunoprecipitation from ectopically expressed TUBB8‐WT and TUBB8‐D417N in ovarian‐derived KGN cells (Figure 5A and B). To gain deeper mechanistic insights into how the D417N missense variant causes oocyte meiotic arrest, we also conducted ultramicro‐quantitative proteomics within WT and D417N knock‐in mouse oocytes. A total of 3408 proteins were identified through proteomic analysis, of which 1121 differentially abundant proteins (828 downregulated and 293 upregulated proteins) were identified between WT and D417N missense variant oocytes (Figure 5C). GO‐enrichment analysis revealed that these downregulated DAPs mainly enriched in terms such as ‘protein binding,’ ‘protein‐containing complex binding’, and ‘catalytic activity, acting on a protein’, implying the D417N missense variant may alter the binding ability of TUBB8 with some key microtubule‐associating proteins, as well as the binding and formation of protein complexes (Figure S8A).

Considering the vast number of GO ontologies, we conducted similarity clustering on the downregulated proteins to explore the key ontology and global functional differences. For the affected molecular function by the D417N missense variant, word clouds illustrated by similarity heatmap indicated that downregulated proteins were significantly enriched in terms including binding, adaptor, and transporter (Figure 5D). These GO terms were found to be involved in various biological processes, including the development, organisation, apoptotic, and migration (Figure S8B). To delve into the functional alterations and differences among the WT and D417N oocytes, we conducted enrichment analyses on the interested and independent subsets of genes significantly downregulated and affected by the D417N missense variant. Specifically, for the organisation cluster depicted in Figure S5B, we observed that the downregulated proteins displayed a diverse and abundant profile related to cell division in their independently enriched GO terms, with a significant emphasis on various spindle assembly processes. These processes include the regulation of actin filament organisation, mitotic spindle assembly checkpoint signalling and meiotic nuclear division, suggesting that the D417N missense variant significantly impacts multiple key processes in the maturation of oocytes (Figure S8C). The network map illustrates the relationships among these enriched BP pathways (Figure S8D). Since tubulin involved in spindle assembly typically functions within microtubule‐associated protein, we conducted a protein‐protein interaction (PPI) analysis on those protein sets to identify key players exhibiting the highest degrees of interaction, thereby emphasising their pivotal roles in these processes (Figure S8E). Additionally, we conducted a comparative analysis of differentially abundant proteins enriched through quantitative proteomics between immunoprecipitation from KGN cells and oocytes. Our findings revealed a high degree of consistency in significantly downregulated proteins between the KGN cell line and oocytes, demonstrating the robustness of our results (Figure 5E), reflecting the similar deleterious effects of the TUBB8‐D417N missense variant across different species.

To further observe the ultramicroscopic structure of the microtubules after the D417N mutation, we used focused ion beam–scanning electron microscopy (FIB‐SEM) and found that the D417N mutated microtubules were short and rich in microtubule breakage, at the same time lacking straight long fibers (Figure 5E). It has been recognised that tubulin undergoes a wide range of PTMs to precisely regulate the physical characteristics—and thus the stability, and dynamics of microtubules in their heterodimeric and protofilament configurations.^47^, ^48^, ^49^ Our KOG‐category analysis revealed that the differentially abundant proteins were primarily associated with processes related to ‘posttranslational modification, protein turnover, chaperones’, ‘signal transduction mechanisms’, and ‘cytoskeleton’ (Figure S8F). Subsequently, we aimed to investigate whether the D417N missense variant affected any PTMs. Microtubule acetylation, a critical factor for stable microtubules and proper dynamics,^49^ was the only affected PTM and was significantly reduced by the D417N missense variant (Figures 5G and H and S9A and B). To further strengthen our findings, we also obtained consistent results using an alternative antibody (Figure S9C).

Interestingly, there was a sharp increase in the expression of HDAC6, a major deacetylase involved in microtubule acetylation (Figure 5H and J). Both EB1 labelling and microtubule acetylation in microtubule dynamics are thought to be regulated by deacetylase HDAC6.^50^, ^51^, ^52^ Co‐immunoprecipitation analysis demonstrated that the affinity of EB1 for microtubules was weakened by the D417N missense variant while its interaction with deacetylase HDAC6 was abnormally increased (Figure S10A). In D417N mutated mouse oocytes, the expression of HDAC6 was also increased together with altered cellular localisation (Figure 5I and J). Moreover, compared to the WT oocytes, a significant decrease in the acetyltransferase αTAT1 was observed in D417N oocytes, potentially disrupting the dynamic balance between microtubule deacetylation and acetylation (Figure S10B–C).

### 3.6 Inhibition of HDAC6 can rescue defects in spindle assembly caused by TUBB8 missense variants

**FIGURE 6:**
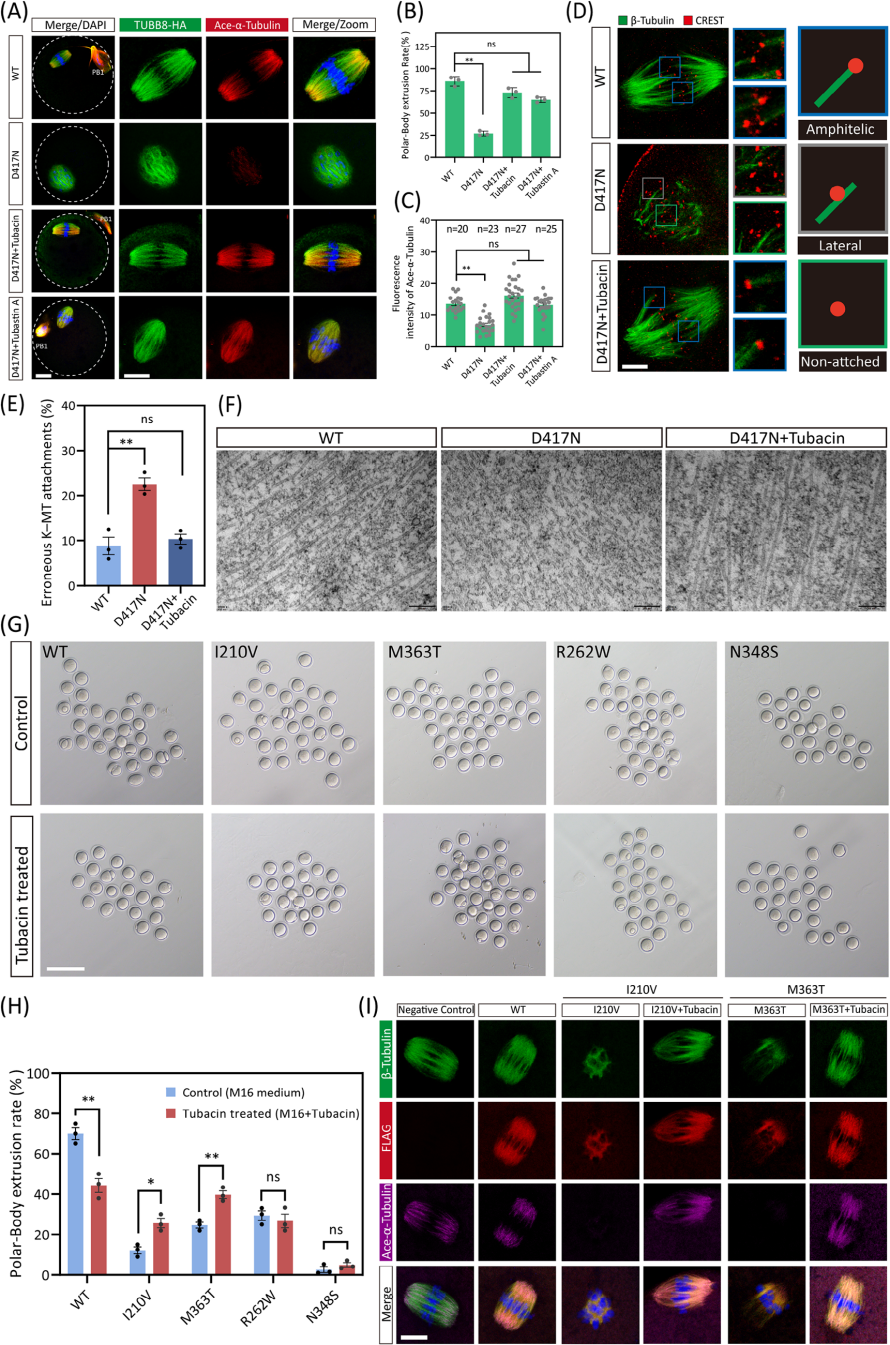
Inhibition of HDAC6 can restore the spindle assembly caused by TUBB8 missense variants. (A) Representative confocal images of spindle morphology in groups of WT, D417N (DMSO), D417N + 2 µM tubacin, and D417N + 10 µM Tubastatin A. Metaphase I oocytes were immunostained with DAPI to visualise chromosomes (blue), TUBB8‐HA to visualise microtubules (green), and ace‐α‐Tubulin (red). (B) Quantitative analysis of PBE rates in mouse oocytes for different groups. D417N oocytes were cultured in M16 medium containing DMSO for 12 h after GVBD; D417N+Tubacin, oocytes were cultured in M16 medium containing 2 µM Tubacin dissolved in DMSO for 12 h after GVBD; D417N+Tubastatin A, oocytes were cultured in M16 medium containing 10 µM Tubastatin A dissolved in DMSO for 12 h after GVBD. Graphs show the mean ± SEM from three independent experiments (One‐way ANOVA with Šidák correction for multiple comparisons, ns = no significance, ** indicates *p* < .01). Numbers indicate the individual oocytes quantified. (C) Quantitative analysis of the mean intensity of ace‐α‐Tubulin. Data were presented as the mean  ±  SEM (One‐way ANOVA with Šidák correction for multiple comparisons, ns = no significance, * indicates *p* < .05, ** indicates *p* < .01). Numbers indicate the individual oocytes quantified. (D) Representative images of K‐MT attachments in WT, D417N, and D417N+2 µM tubacin oocytes. K‐MT is mainly divided into amphitelic attachments, merotelic attachments, and lateral interactions. Scale bar, 10 µm. (E) Graph shows the average percentage of centromeres without cold‐stable attachments. Error bars represent SEM for three independent experiments (> 100 bivalents analysed in each experiment). One‐way ANOVA with Šidák correction for multiple comparisons, ** indicates *p* < .01, ns = no significance. (F) Transmission electron microscopy revealed that microtubules in D417N oocytes supplemented with Tubacin exhibited longer extensions, indicating a more stable state. Scale bars, 200 nm. (G) Representative light microscopy images of PBE in WT and mutants of TUBB8 oocytes after GVBD 12 h. GV oocytes were microinjected with TUBB8‐WT mRNA (200 ng/µL) or missense variants mRNA (200 ng/µL), and maintained in IBMX for 20 h before being washed into IBMX‐free M16 medium or Tubacin‐treated medium (M16 medium +Tubacin) to allow resumption of meiosis. Scale bar, 200 µm. (H) Quantification of the percentage of oocytes in (F) that undergo PBE in vitro. Data were presented as the mean  ±  SEM from three independent experiments (Unpaired Student's *t*‐test, two‐tailed, *denotes *p* < .01, and ** denotes *p* < .05). The total number of oocytes used for statistical analysis in WT (*n* = 100), I210 V (*n* = 90), M363T (*n* = 96), R262 W (*n* = 85), N348S (*n* = 78). (I) Representative immunofluorescence images of mouse oocytes microinjected with Flag‐tagged RNA encoding WT or missense variants of TUBB8. Green, β‐tubulin; red, FLAG (TUBB8); magenta, acetylated microtubule (ace‐α‐Tubulin); blue, DNA (DAPI). Scale bars, 20 µm.

Subsequently, we proposed that inhibiting the excess HDAC6 deacetylase may help restore the microtubules and spindle in oocytes with the D417N missense variant. Supporting this hypothesis, we found that treatment with Tubacin, a selective inhibitor of HDAC6, reestablished the microtubule acetylation level, the bipolar spindle morphology, and even the polar body extrusion rates in D417N mutant oocytes (Figure 6A–C). Similarly, successful rescue results were observed incubating with Tubastatin A, an alternative HDAC6‐specific inhibitor (Figure 6A–C). These results suggest that appropriate suppression of HDAC6 is crucial for proper microtubule stabilisation and spindle assembly to rescue D417N oocytes. We also tested the occurrence of aberrant spindle/chromosome structure to evaluate the kinetochore‐microtubule interaction. The abnormal K‐MT attachments were considerably more in D417N missense variant oocytes compared with controls but were significantly declined after Tubacin supplementation (Figure 6D and E). Furthermore, the results from transmission electron microscopy revealed that microtubules in D417N oocytes supplemented with Tubacin exhibited longer microtubule fibers similar to WT, indicating a more stable state.

We also found that the inhibition of HDAC6 using Tubacin resulted in a sharp increase of EB1 expression in cultured cells (Figure S10D). Considering the decreased affinity between EB1 and TUBB8 leading to microtubule instability, we wondered if other microtubule polymerisation‐promoting chemicals could have similar rescue effects. Nonetheless, treatment with Taxol, a classical microtubule stabiliser, did not improve spindle assembly in D417N‐mutated oocytes, indicating that accelerated nucleation alone is insufficient for bipolar spindle formation (Figure S7C). In addition, supplementation of PCI‐34051 (a specific HDAC8 inhibitor) to D417N mutant oocytes did not improve either microtubule stability or barrel‐shaped spindle assembly (Figure S10E).

Furthermore, we found that the abnormally increased BuBR1‐based activation of the spindle assembly checkpoint (SAC) was also corrected by the supplement of Tubacin, indicating that the metaphase I to anaphase I transition failure caused by the D417N missense variant was rescued (Figure S11A and B). Furthermore, no increase in chromosomal aneuploidy rate was observed following the rescue of Tubacin (Figure S11C and D). Time‐lapse imaging also revealed normal microtubule nucleation, spindle assembly, and chromosome segregation in D417N oocytes upon supplementation with Tubacin supplementation (Figure S11E and F).

To determine whether the rescue strategy with HDAC6 inhibitors can be expanded in other TUBB8 pathogenic variants, we microinjected mRNA carrying the TUBB8 missense variants that do not affect a/β‐tubulin heterodimer yields into mouse GV oocytes and maintained them in M2 medium with IBMX for 20 h. Subsequently, the oocytes were cultured with IBMX‐free medium with or without Tubacin to allow resumption of meiosis, and the progression of oocyte maturation was analysed. We observed that Tubacin treatment could rescue a proportion of TUBB8 missense variants, including I210 V and M363T, as a significant increase in the polar body extrusion rate was found (Figure 6G and H). Moreover, successful assembly of bipolar spindles was observed following Tubacin treatment, accompanied by recovery of microtubule acetylation levels in the I210 V and M363T mutants (Figure 6I).

In addition, to further validate the rescue effect of HDAC6 inhibitors on TUBB8 missense variants, we transfected HeLa cells with FLAG‐tagged WT and mutant TUBB8 constructs. Consistent with previous studies,^4^, ^38^ the majority (85%) of cells expressing TUBB8‐WT exhibited normal microtubule morphology characterised by co‐assembly of β‐tubulin (TUBB8) and α‐tubulin into a well‐organised network, however higher percentages of abnormal and obliterated microtubule network were observed in TUBB8 mutants bearing cells (Figure S12A). Of note, the microtubule network was almost unaffected in TUBB8‐WT‐expressing cells by Tubacin treatment (Figure S12A–C). However, in the I210 V and M363T missense variants with abnormal microtubule networks, Tubacin treatment partially restored their disrupted microtubule, resulting in an increase in the percentage of normal microtubule networks from 23.4% (control) to 38.5% (Tubacin treated) for I210 V and from 31.7% (control) to 45.2% (Tubacin treated) for M363T (Figure S9B). These findings prove that certain TUBB8 missense variants can be partially rescued by selective HDAC6 inhibitors.

Collectively, these results suggested that reduced microtubule acetylation in D417N oocytes was caused by upregulation of HDAC6, and inhibition of HDAC6 could rescue meiotic arrest in D417N, I210 V and, M363T mutant oocytes.

### 3.7 Post‐translational modification profiling of TUBB8 and structure prediction for TUBB8 orthologs

The specific expression of TUBB8 in oocytes and early embryos of both human and higher non‐human primates remains unexplained. The spatiotemporal regulation of tubulin post‐translational modifications is crucial for modulating microtubule properties and may be involved in the regulation of oocyte maturation. Thus, we comprehensively analysed published data on post‐translational modifications of α/β‐Tubulin in human and mice. We found that TUBB8 harbours extensive phosphorylation, ubiquitination, palmitoylation, and mono‐methylation (Figure S13A), similar to other β‐tubulin isotypes. However, we observed that TUBB8 lacks SUMOylating, O‐glycosylation, and Di‐methylation, which may be partly attributed to the limited research on TUBB8 at present (Figure S13A). Furthermore, in contrast to the variability observed in human microtubule PTMs, the mouse PTMs of α and β tubulin isotypes exhibit a relatively consistent pattern (Figure S13B).

Furthermore, to identify potential unique post‐translational modifications on TUBB8, we conducted a PTMs‐omic profiling via TUBB8‐WT and TUBB8‐D417N IP‐MS in HEK293T cell line model. Surprisingly, our findings revealed that TUBB8 harbours several novel post‐translational modifications. These modifications encompassed malonylation, succinylation, benzoylation, glutarylation, propionylation, lactylation, and crotonylation (Figure S13C). The D417N mutation was found to affect the PTMs of TUBB8 and its interacting proteins. Specifically, the D417N mutation resulted in a slight decrease in the phosphorylation level of TUBB8. Additionally, it led to a reduction in the malonylation of NUP155, a nuclear pore complex protein that interacts with TUBB8. Furthermore, the phosphorylation level of the ciliary microtubule dynein DNAH10 was also decreased.

Mouse tubulin isoforms were predicted with Alphafold2 for the structural similarity to the human TUBB8 protein. The results revealed that mouse TUBB4B, TUBB4A, and TUBB2B exhibited the highest sequence identity to TUBB8 (Figure S14A), while TUBB2B displayed highest structural similarity than TUBB4B and TUBB4A (Figure S14B). It was noted that the tail coiled‐coil structures of these orthologs differed significantly from that of TUBB8, supporting the theory that the functional diversity of tubulin isotypes is largely influenced by their C‐terminal tails.^53^ This finding potentially highlights a unique feature of the human TUBB8 protein. Taken together, these results demonstrated the structural similarity to TUBB8 from its murine homologs, and also the divergence in the coiled‐coil tail region, which may have important implications for understanding the functional specificity of TUBB8 in human.

## 4 DISCUSSION

Female infertility resulting from TUBB8 missense variants encompasses a broad range of pathogenic missense variants associated with oocyte meiotic arrest. Previous data indicated that partial TUBB8 missense variants could disrupt the de novo assembly of heterodimers or their binding to tubulin cofactors. However, the mechanisms of other missense variants with unaffected yields of α/β‐tubulin heterodimers resulting in ruined spindle remain unclear. Revealing the precise etiology of spindle defects may contribute to the development of therapeutic strategies to restore oocyte maturation in human eggs and enhance outcomes of assisted reproductive technology.

**FIGURE 7:**
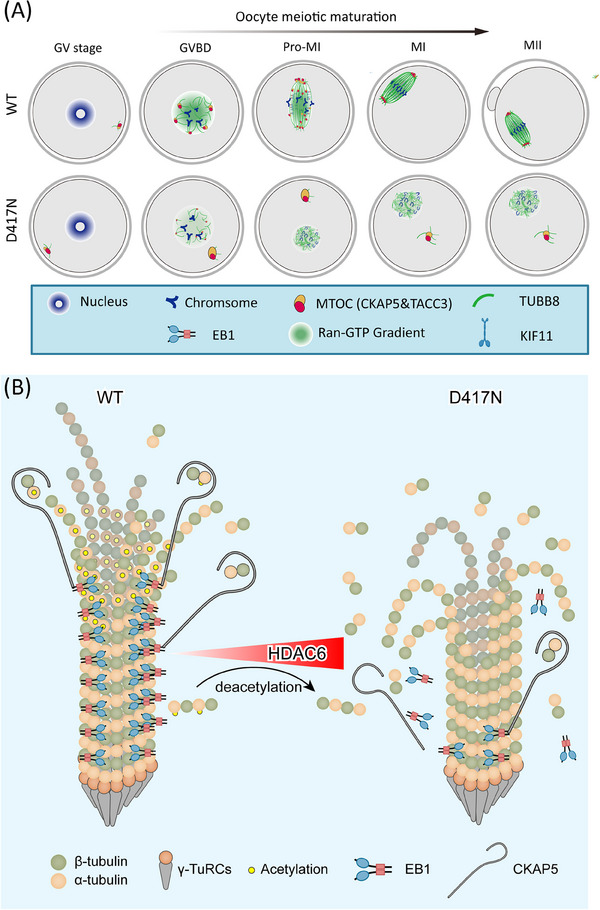
Schematic representation of proposed mechanisms in TUBB8 D417N oocytes with a meiotic arrest phenotype. (A) In wild‐type oocytes, CKAP5 assembles near the cortex of GV oocytes and fragments to the nuclear envelope after GVBD, which then recruits and initiates microtubule nucleation for meiotic spindle assembly. However, in oocytes with the D417N missense variant of TUBB8, spindle assembly during meiosis I is compromised due to the inability to recruit CKAP5 and aberrant microtubule nucleation resulting from decreased affinity between TUBB8 and EB1 (described in B in detail). (B) Working model illustrating the underlying mechanism of perturbation of microtubule nucleation and assembling the spindle by D417N missense variant. The growth and stability of the microtubule are mediated by the key role of EB1 at the growing microtubule plus ends, while the microtubule is acetylated as the stable microtubule. CKAP5 specifically binds to free tubulin heterodimers and facilitates their concentration at the plus ends, promoting microtubule polymerisation. Mechanistically, the reduced affinity of EB1 due to the D417N missense variant impairs its ability to track along microtubule plus ends and stabilise them, consequently disrupting the dynamic interaction between microtubule plus ends and CKAP5 that is essential for initiating microtubule nucleation and spindle assembly. Furthermore, diminished binding of EB1 to microtubules leads to decreased stability as indicated by reduced acetylation levels; however, in contrast, the presence of EB1 inhibits HDAC6 deacetylase activity thereby promoting increased acetylation levels and enhancing microtubule stability.

To elucidate the deleterious effect of the human TUBB8 pathogenic variants in vivo, we generated oocyte‐specific knock‐in mouse models that mimic the equivalent human missense variants. Our study elucidated the puzzle regarding the clear mechanistic models for how unaffected yields of a/β‐tubulin heterodimer missense variants in TUBB8 result in defective spindle assembly. We discovered that mutant TUBB8 tubulin heterodimers intrinsically disrupted the interaction between EB1 and microtubules, resulting in microtubule instability and ultimately driving the delocalisation of CKAP5, a key microtubule nucleator, thereby dysregulating microtubule dynamics and perturbing spindle assembly as well as oocyte maturation arrest. Diminished affinity between EB1 and microtubules compromised their stability as indicated by reduced levels of acetylated tubulin. Therefore, we proposed a potential therapeutic strategy involving inhibiting HDAC6 activity in affected TUBB8 mutant oocytes. Collectively, these findings provide comprehensive insights into the regulation mediated by EB1, CKAP5 and acetylation impairing microtubule nucleation and spindle assembly during oocyte meiotic maturation (Figure 7), and the rescue effects of HDAC6 inhibition treatment in TUBB8 missense variant mouse oocytes.

It is worth noting that a recent study has demonstrated that the ectopic expression of TUBB8 resulted in mouse oocyte aneuploidy, and female mice ectopically expressing human TUBB8 were completely infertile.^54^ In their mouse model, ZP3‐promoter was directly introduced into the Rosa26 loci, together with TUBB8 coding sequencing, which is an unusual strategy for mouse model construction. However, our wild‐type TUBB8 mouse model was established in a different way, and we found that oocytes matured normally. The typical Cre‐loxP system was used mediated by CRISPR‐Cas9 to knock‐in wild‐type and pathogenic missense variant TUBB8, and subsequently crossed with Gdf9‐cre mice to generate oocyte‐specific knock‐in mouse model. We supposed that the infertility of TUBB8‐WT female mice in the previously mentioned study was caused by the high ectopic expression from the strong Zp3 promoter. Although the whole complicated role of TUBB8 in female infertility has not been fully understood, it is acceptable to have the traditional Cre‐loxP TUBB8‐WT knock‐in mice with normal oocyte maturation as the control group in the current study to explore the mechanisms of the D417N mutation impairing the oocyte meiotic process.

The assembly of the spindle, primarily mediated through cytokine‐microtubule nucleation and microtubule‐associated proteins, constitutes the central event in oocyte meiosis. EB1, a plus end‐tracking protein (+TIP), has been proposed to act as a stabiliser for microtubules and accumulate at growing microtubule ends, thereby playing a crucial regulatory role in microtubule dynamics.^55^ It is widely acknowledged that oocytes lack centrosomes and instead utilise aMTOCs to facilitate microtubule nucleation and the formation of a bipolar spindle in rodent mammals.^20^ CKAP5, known for its role in promoting microtubule polymerisation by binding free tubulin near growing plus ends, has also been identified as a crucial factor downstream of EB1 for microtubule nucleation.^39^ Interestingly, our findings demonstrate colocalisation of CKAP5 and Pericentrin, a well‐known component of aMTOCs, suggesting that CKAP5 serves as an indicator of the presence of aMTOCs. Before GVBD, CKAP5 localises at the cortex of germinal‐vesicle oocytes and subsequently migrates to the nuclear membrane before its rupture (Figure 7A). Until GVBD, CKAP5 is recruited to initiate microtubule nucleation until GVBD occurs; it then fragments into multiple small‐sized aMTOCs, which are later sorted and re‐clustered at the two poles of the bipolar spindle (Figure 7A). Remarkably, this three‐step process of spindle assembly aligns with recent reports on huoMTOC‐guided microtubule nucleation in human oocytes where canonical pericentriolar material pericentrin is absent.^22^, ^43^ Therefore, it is likely that the function of CKAP5 is conserved in terms of microtubule nucleation and spindle assembly between human and rodent mammalian oocytes. In D417N oocytes, CKAP5 exhibited an abnormal unfragmented localisation at the nuclear periphery rather than the perinuclear region throughout the microtubule nucleation and spindle assembly stages, resulting in defective spindle assembly ultimately due to a loss of affinity with EB1 during microtubule nucleation. Besides CKAP5, the localisation of TACC3 in D417N oocytes also underwent alterations during the microtubule nucleation stage, resulting in the absence of the unique LISD structure at meiosis I; however, TACC3 expression itself did not hinder this process. Previous studies have suggested that TACC3 plays a crucial role as a core component for microtubule growth and spindle assembly in centrosomal spindles.^56^, ^57^ Impairment of TACC3 expression in ovarian cancer cells leads to blocked microtubule nucleation and impaired spindle assembly.^58^ In a recent study, a distinct LISD structure of meiotic spindle assembly was identified in mouse oocytes where TACC3 serves as a central component for regulating critical factors required for stable meiotic spindle assembly.^40^ These findings suggest that the D417N missense variant disrupts specific regulatory mechanisms typically mediated by CKAP5, thereby causing oocyte maturation arrest.

Microtubules undergo various PTMs that regulate the properties and functions of the microtubule cytoskeleton. Acetylated tubulin serves as a marker for stable microtubule polymerisation and exhibits high localisation in mouse oocytes, contributing to the formation of stable bipolar spindles.^59^ The acetylation process is tightly regulated by the balanced activities of acetyltransferases and deacetylases, with HDAC6 and HDAC8 primarily responsible for deacetylation in oocytes.^60^, ^61^, ^62^ Our data demonstrated a significant reduction in microtubule acetylation levels in D417N mutant oocytes compared to WT oocytes, accompanied by an upregulation of the deacetylase HDAC6. Moreover, inhibition of HDAC6 not only rescued phenotypes associated with the D417N missense variant but also reduced defects caused by other TUBB8 variants such as I210 V and M363T, exhibiting substantial improvements upon suppression of HDAC6 activity. Emerging evidence suggests that EB1 labelling and microtubule acetylation are regulated by HDAC6 during dynamic microtubule events.^52^ Furthermore, accumulation of HDAC6 leading to excessive microtubule deacetylation has been observed in various diseases, including lung cancer^63^ and neurodegenerative disorders.^64^, ^65^, ^66^, ^67^, ^68^ Treatment with selective inhibitors targeting HDAC6 significantly improves Alzheimer's disease phenotypes, highlighting its potential as a promising therapeutic target warranting further investigation. Numerous studies have demonstrated that inhibition of HDAC6 activity exhibits promising anticancer efficacy in various tumours, including breast cancer and ovarian cancer.^69^, ^70^ Although our study focused on treating oocytes carrying missense variants rather than directly treating patients, upon further investigation of safety and efficacy, it may be plausible to administer treatment to patients using liposomes containing HDAC6 inhibitors as potential therapeutic interventions in the future. It is noteworthy that clinical trials have demonstrated that HDAC6 inhibitors, such as ACY1215,^71^ are well‐tolerated and exhibit minimal toxicity. Consequently, our research offers a scientific rationale for the potential clinical translation of these findings through the repurposing of clinically applicable HDAC6 inhibitors for the treatment of patients harbouring the D417N mutation, for which no effective therapies are currently available.

In this study, we propose a model whereby the D417N missense variant intrinsically alters EB1 regulation, which perturbs CKAP5 localisation and dysregulates microtubule nucleation. Our findings also reveal that the instability of assembled microtubules and decreased acetylation levels are common mechanisms underlying spindle assembly defects. Mutant oocytes can be partially recovered upon Tubacin treatment, exhibiting normal spindle morphology and polar‐body extrusion. In summary, our study characterises a comprehensive mechanism for TUBB8 missense variants responsible for human oocyte maturation arrest at metaphase I, thus opening new avenues for clinical interventions in treating female infertility.

## AUTHOR CONTRIBUTIONS

Hui Luo and Jianhua Chen contributed equally to this work. Hui Luo, Jianhua Chen, and Ruizhi Feng conceived and designed the experiments and analyses; Hui Luo performed all experiments and analysed the data with the following exceptions: Tian Wu, Siyue Yin, and Guangping Yang performed microinjection mRNA in oocytes; Hui Luo, Jianhua Chen, and Ruizhi Feng drafted and revised the manuscript; Cao Li performed validation experiments for these studies. Yipin Wang, Zhihan Guo, Saifei Hu, Yanni He, Yingnan Wang, Yao Chen, Congxiu Miao, and Yun Qian performed formal analyses of the data in this study. Youqiang Su provided resources for these studies.

## CONFLICT OF INTEREST STATEMENT

The authors declare no conflicts of interest.

## ETHICS STATEMENT

All animals were maintained and used according to the guidelines of Institutional Animal Care and Use Committee of Nanjing Medical University, with the ethical approval number IACUC‐1912016. All animal procedures were approved by the Institutional Animal Care and Use Committee of Nanjing Medical University and were conducted following the institutional guidelines for the Animal Care and Use Committee of Nanjing Medical University.

## Supporting information



Supporting Information

## Data Availability

All data are available in the main text or the Supplementary Materials.
