# Supplementary material for: Pathogenic variants of TUBB8 cause oocyte spindle defects by disrupting with EB1/CAKP5 interactions and potential treatment targeting microtubule acetylation through HDAC6 inhibition
Source: Clin Transl Med. 2025 Jan 20;15(1):e70193. doi: 10.1002/ctm2.70193 (PMC11746963; doi:10.1002/ctm2.70193)
Supplement: Supplementary file 1 — Supporting Information [file CTM2-15-e70193-s001.pdf]

Pathogenic variants of TUBB8 cause oocyte spindle defects by disrupting with EB1/CAKP5 interactions and potential treatment targeting microtubule acetylation through HDAC6 inhibition

Hui Luo[1], Jianhua Chen[1], Cao Li[1], Tian Wu[1], Siyue Yin[1], Guangping Yang[2], Yipin Wang[1], Zhihan Guo[1], Saifei Hu[1], Yanni He[1], Yingnan Wang[1], Yao Chen[3], Youqiang Su[4], Congxiu Miao[5], Yun Qian[3], Ruizhi Feng [1,3,6*]

SUPPORTING Information

Figures S1–S14.

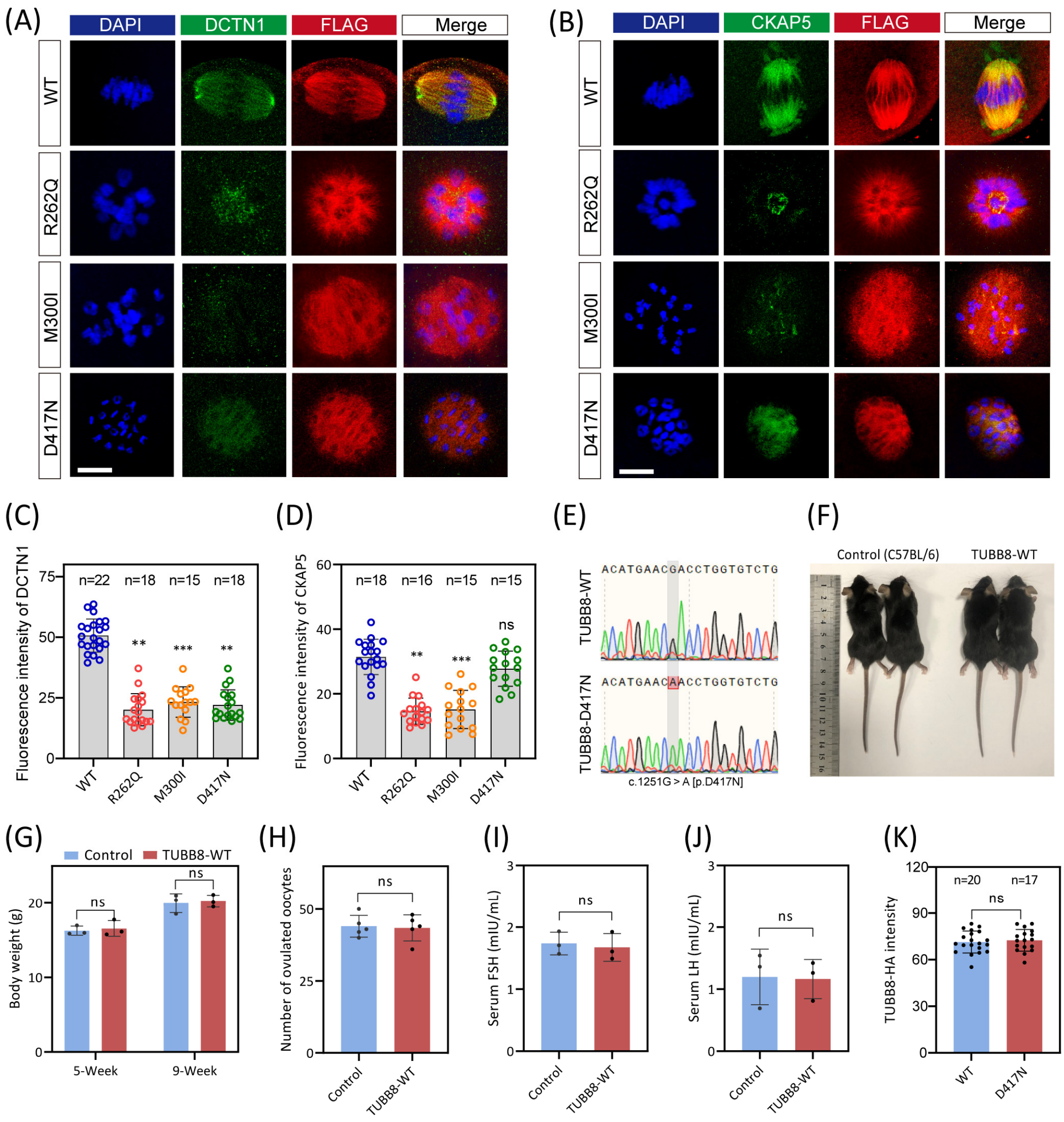

Figure S1. Impaired DCTN1 and CKAP5 activity observed in oocytes carrying TUBB8 mutations. (A) Representative immunofluorescence images of mouse oocytes microinjected with Flag-tagged RNA encoding wild-type (WT) or mutant forms of TUBB8 are shown. Metaphase I oocytes were then immunostained with DAPI to visualize chromosomes (blue), FLAG to visualize TUBB8 expression (red), and DCTN1 labeling growing microtubules (green). Scale bars, 10 µm. (B) Metaphase I oocytes were immunostained with DAPI to visualize chromosomes (blue), FLAG to visualize TUBB8 expression (red), and CKAP5 labeling microtubule nucleation (green). Scale bars, 10 µm. (C-D) Statistical analysis of mean intensity of DCTN1 (C) and CKAP5 (D) in mouse oocytes expressing different TUBB8 mutations. Numbers

indicate the number of oocytes quantified. P-values were calculated using One-way ANOVA with Šidák correction for multiple comparisons to panels C and D (**denotes P<0.01, *** indicates P<0.001, ns=no significance). (E) Chromatogram of Sanger sequencing illustrating the D417N missense point mutation of TUBB8. (F-G) TUBB8-WT knockin mice exhibited normal physiological functions (F), maintained stable body weights (G), ovulated normal oocytes(H), serum FSH level (I), serum LH level (J). (K) Quantification of mean TUBB8-HA fluorescence intensity for WT and D417N oocytes (Unpaired Student's t-test, two-tailed, ns=no significance).

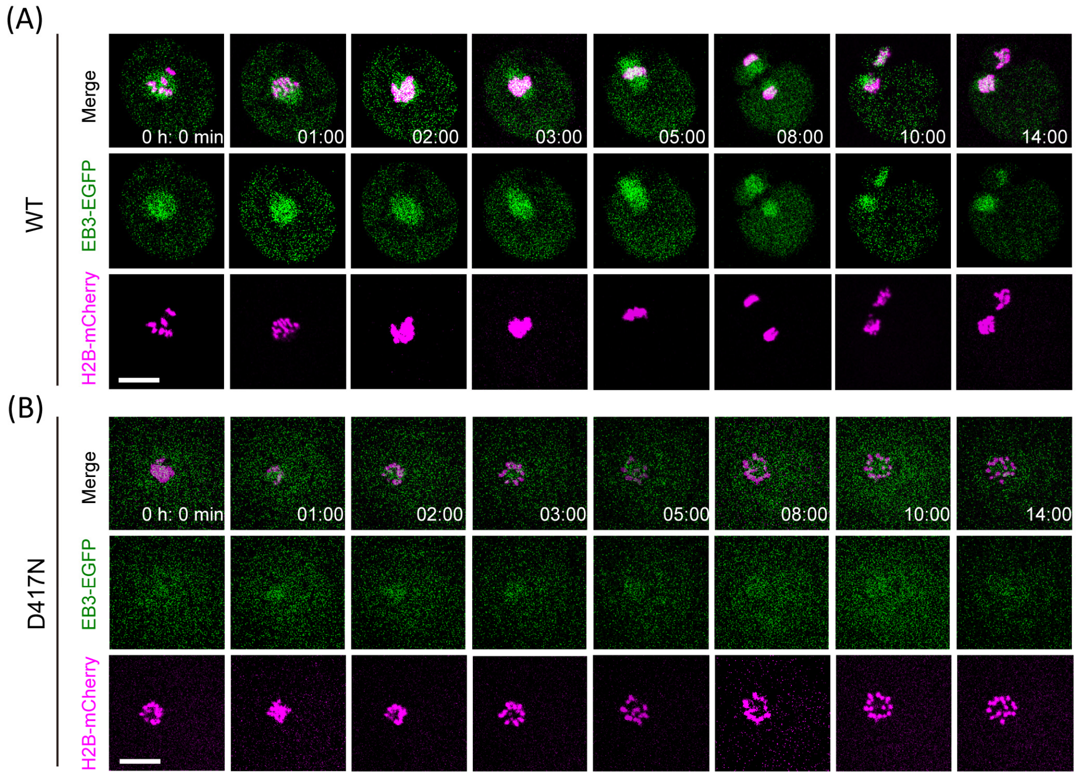

Figure S2. Impaired EB3 activity observed in oocytes carrying TUBB8-D417N mutation. (A-B) Time-lapse imaging of a maturing oocyte expressing EB3-EGFP (microtubule plus ends, green) and H2B-mCherry (chromosomes, magenta) in WT (A) and D417N (B) oocytes. The scale bar is 25 μm. Time hh: mm relative to GVBD.

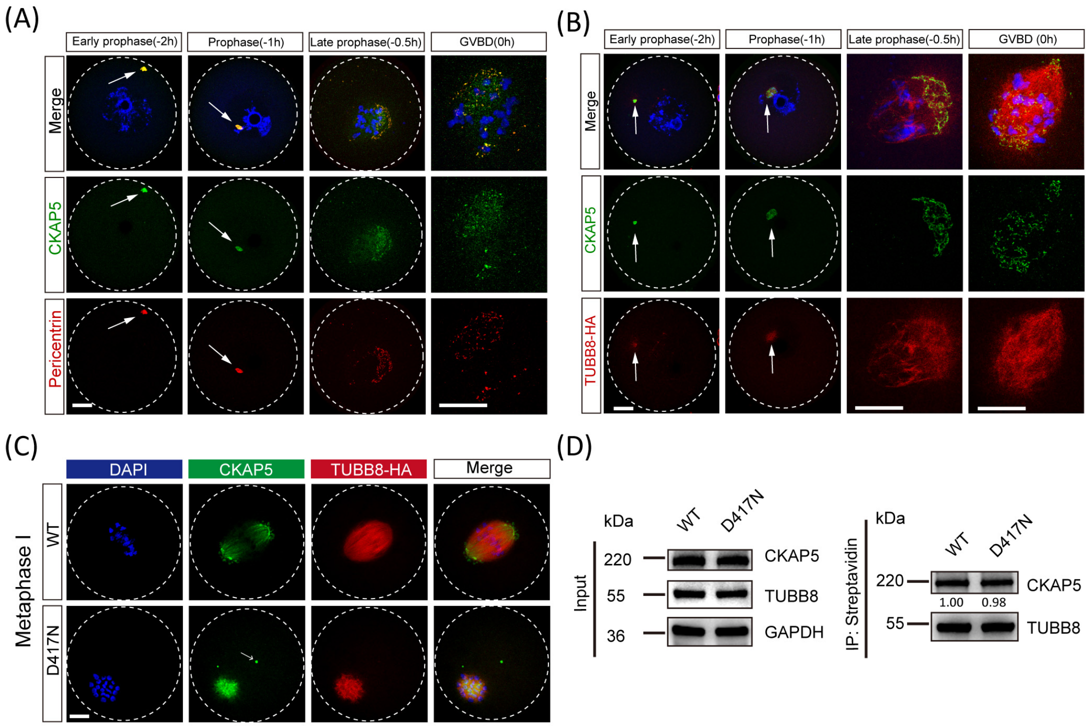

Figure S3. Representative immunofluorescence images of the CKAP5 colocalization with pericentrin and microtubule. (A) Representative immunofluorescence images of the CKAP5 colocalization with pericentrin in mouse oocytes at early prophase (GV stage, -2h), at prophase (-1h), late prophase (-0.5h) before GVBD , and at GVBD (0h). Green, CKAP5; red, MTOC (pericentrin); blue, DNA (DAPI). White arrows indicate the colocalization of CAKP5 and pericentrin. The dashed line demarcates the oocyte. Time is given as ~hours relative GVBD. Scale bar, 10 µm. (B) Representative immunofluorescence images of the CKAP5 localization in mouse oocytes at early prophase (GV stage, -2h), at prophase (-1h), late prophase (-0.5h) before GVBD, and at GVBD (0h). Green, CKAP5; red, microtubule (TUBB8-HA); blue, DNA (DAPI). White arrows indicate the colocalization of CAKP5 and microtubule. The dashed line demarcates the oocyte. Time is given as ~hours relative GVBD. Scale bar, 10 µm. (C) Representative immunofluorescence images of mouse oocytes at metaphase I. Green, TACC3; red, microtubule (TUBB8-HA); blue, DNA (DAPI). The dashed line demarcates the oocyte. Scale bar, 10 µm. (D) Co-immunoprecipitation was performed to determine the affinity between TUBB8 and CKAP5 by D417N mutation. The blots of protein precipitants were probed with CKAP5, GAPDH, and streptavidin (TUBB8) antibodies. GAPDH serves as loading control.

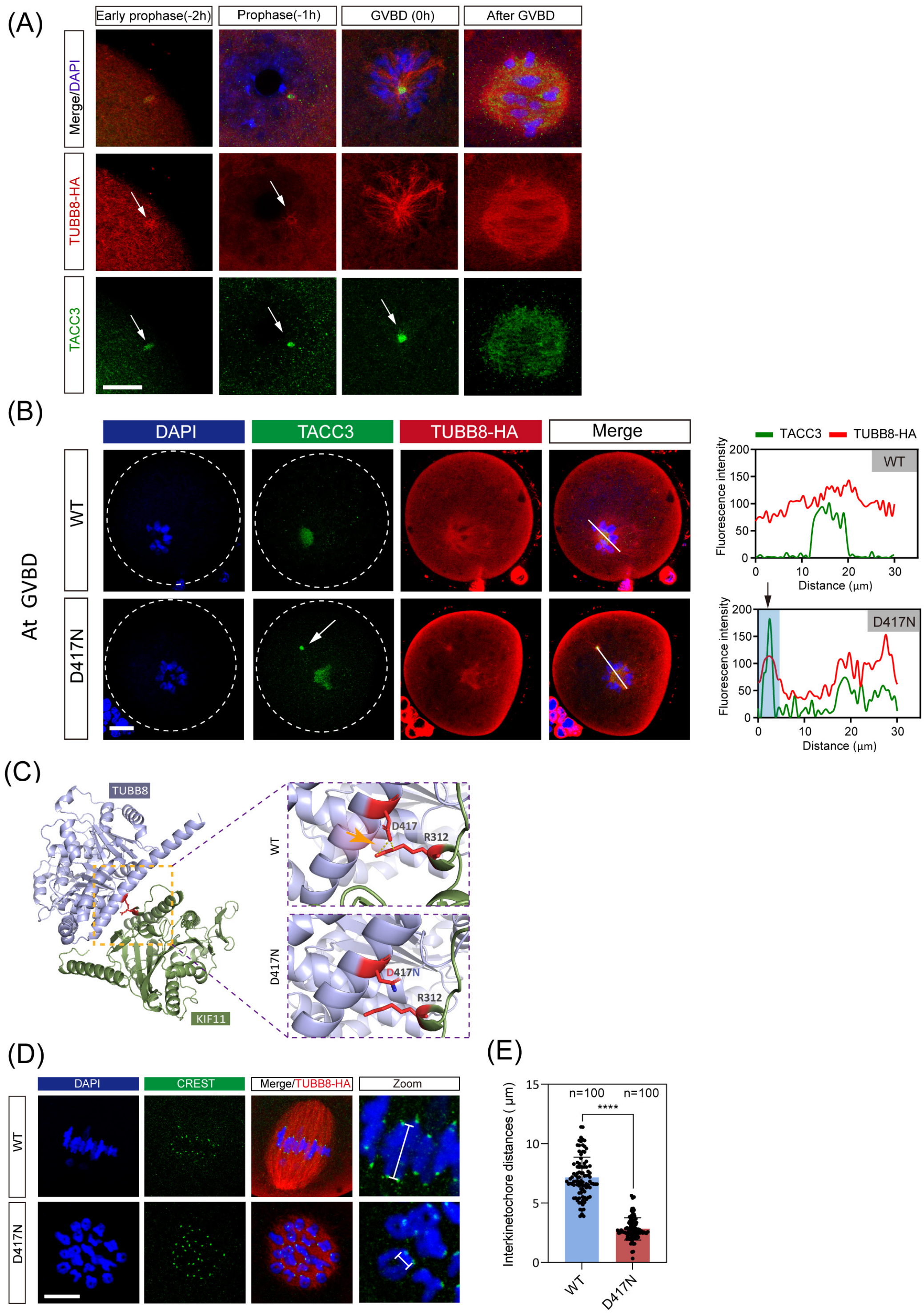

Figure S4. Representative immunofluorescence images of the TACC3. (A) Representative immunofluorescence images of the CKAP5 localization in mouse oocytes at early prophase (GV stage, -2h), at prophase (-1h), late prophase (-0.5h) before GVBD, at GVBD (0h), and after GVBD (1h). Green, CKAP5; red, microtubule (TUBB8-HA); blue, DNA (DAPI). White arrows indicate the colocalization of CAKP5

and the microtubule. Scale bar, 10 μm. (B) Representative immunofluorescence images of mouse oocytes at GVBD. Green, TACC3; red, microtubule (TUBB8-HA); blue, DNA (DAPI). Intensity profiles along the white lines are shown in right panels for oocytes at GVBD. Scale bar, 10 μm. The dashed line demarcates the oocyte. Black arrows indicate that TACC3 is asymmetrical in D417N oocytes, whereas it is even distributed around the chromosome in WT oocytes. (C) The predicted structure between TUBB8 and KIF11 by AlphaFold2 and visualization by Pymol. (D) Representative images of MI spindles in WT and D417N mutation oocytes. Green, CREST; red, microtubule (TUBB8-HA); blue, DNA (DAPI). Scale bar is 10 μm. (E) Statistical analysis of the interkinetochore distances. Data were presented as the mean ± SEM. Numbers indicate the number of bivalents quantified. **** $P < 0.0001$ by Unpaired Student's t-Test.

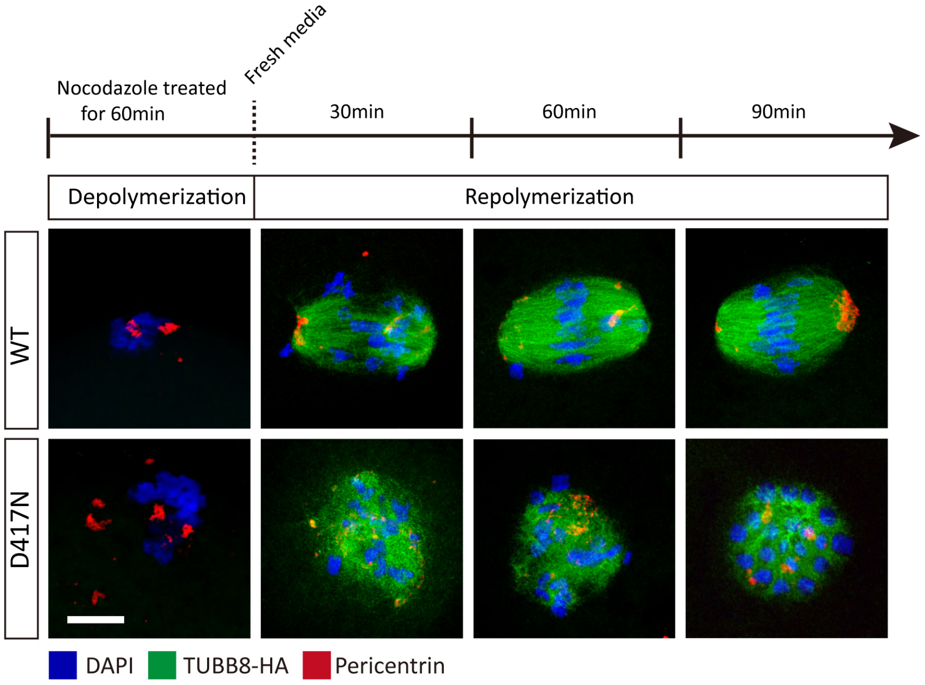

Figure S5. Microtubule depolymerize and regrowth. Immunofluorescence images of mouse oocytes fixed at different times after nocodazole treatment for 60min and washout for 30min, 60min and 90min. Green, microtubules (TUBB8-HA); blue, DAPI (Chromsome); red, aMTOCs (pericentrin). Scale bar, 10 μm.

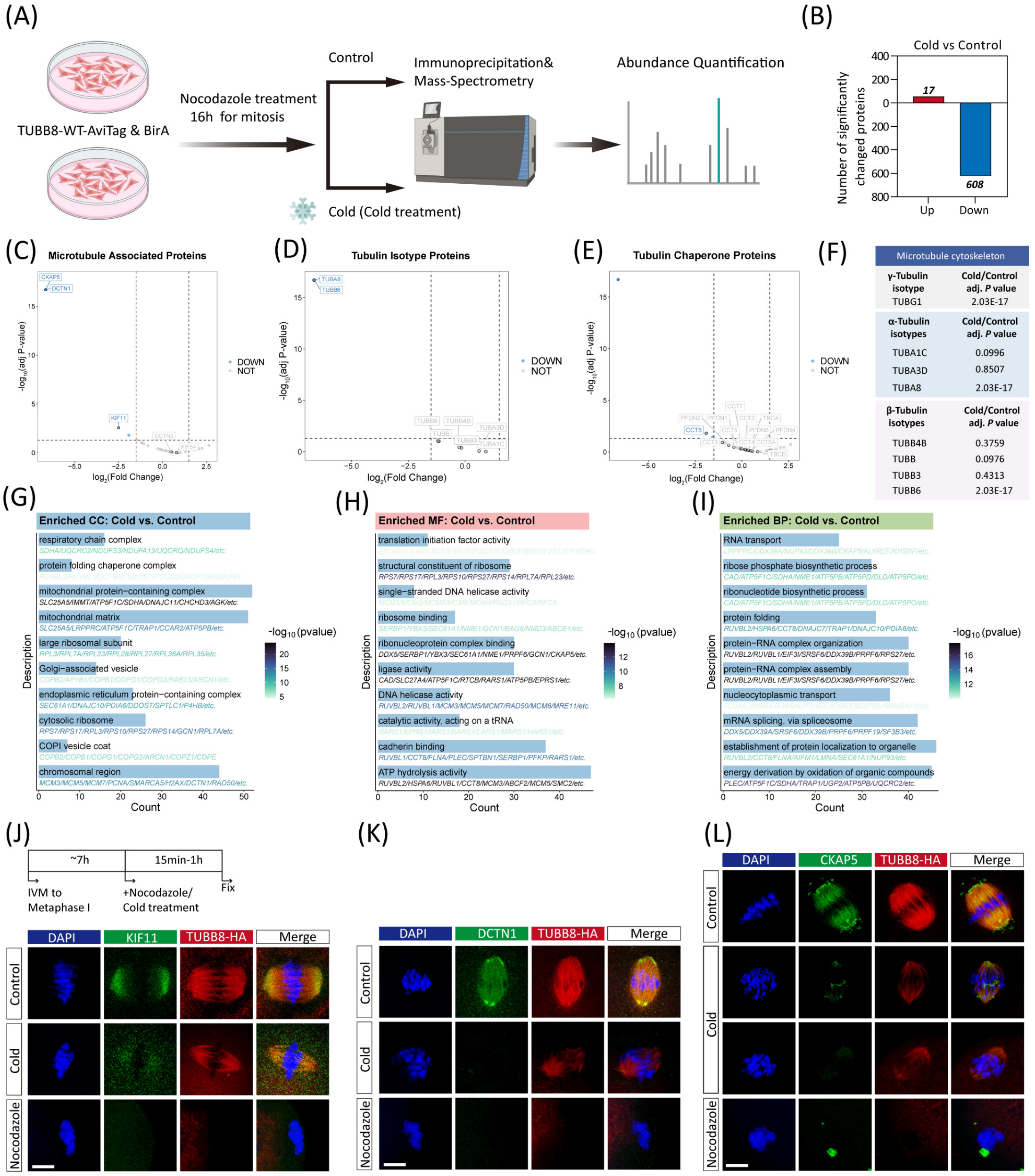

Figure S6. Identification of microtubule-associated factors tightly binding with TUBB8. (A) Immunoprecipitation-mass spectrometry was employed to identify proteins that exhibited significant differences following induction through cold treatment. (B) The bar graph indicates the number of differentially abundant proteins between Control and Cold. (C-E) Control and Cold treatment (Cold) samples were utilized for mass spectrometry and proteome analysis of microtubule-associated protein (C), tubulin isotypes (D), or tubulin chaperone (E). (F) The differentially abundant microtubule isotypes between Control and Cold. (G-I) gene ontology (GO) pathway enrichment analysis with decreased abundant proteins in CC (Cell Component) (G),

MF(Molecular Function) (H), and BP(Biological Process) (I). (J) Immunofluorescence images of mouse metaphase I oocytes treated with cold or nocodazole. Green, KIF11; red, microtubules (TUBB8-HA); blue, chromosomes (DAPI). (K) Immunofluorescence images of mouse metaphase I oocytes treated with cold or nocodazole. Green, DCTN1; red, microtubules (TUBB8-HA); blue, chromosomes (DAPI). (K) Immunofluorescence images of mouse metaphase I oocytes treated with cold or nocodazole. Green, CKAP5; red, microtubules (TUBB8-HA); blue, chromosomes (DAPI). Scale bar, 10 μm.

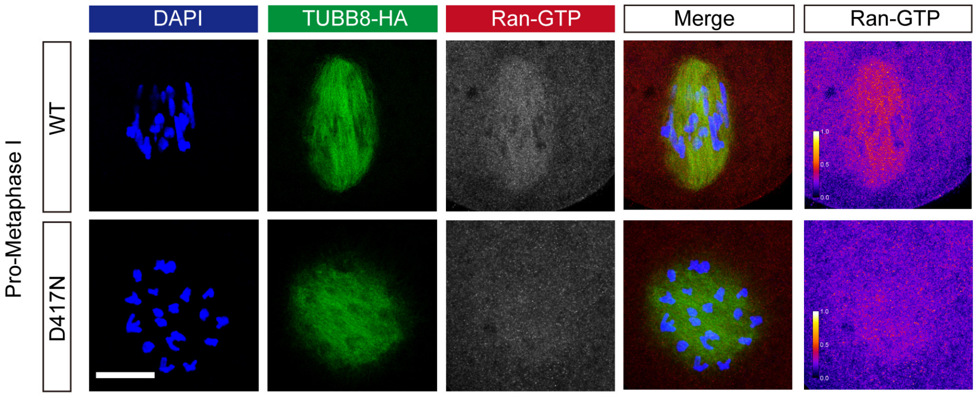

Figure S7. Representative immunofluorescence images of mouse oocytes at Pro-MI stages of meiosis. Green, microtubule (TUBB8-HA); red, active-Ran (Ran-GTP); blue, DNA (DAPI). Right panels showing LUT color grading according to intensity of the Ran-GTP. Scale bar, 10 μm.

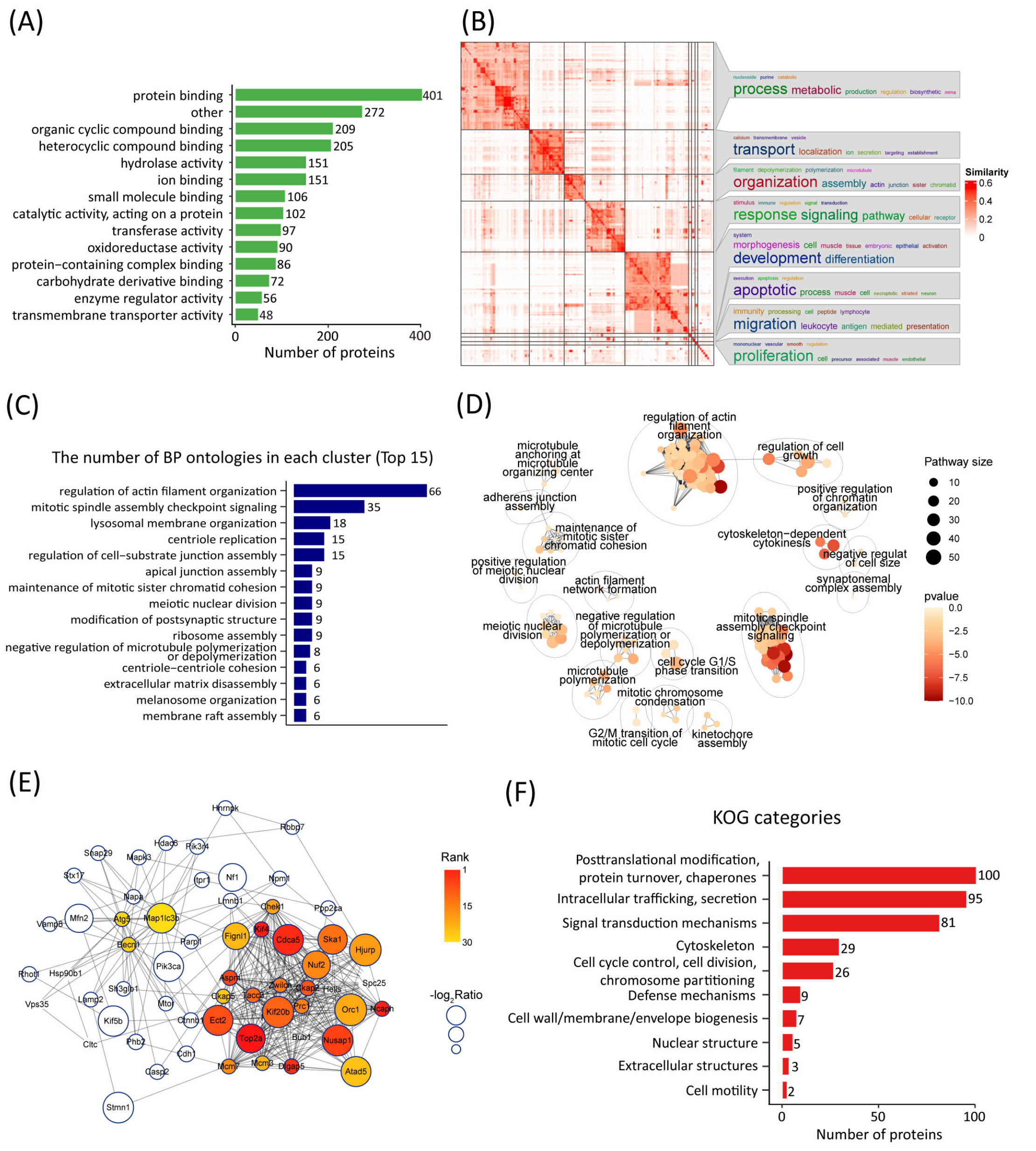

Figure S8. Dysregulated proteins and biological processes in D417N mutation oocytes. (A) GO terms of down-regulated proteins in D417N missense variant oocytes. (B) Similarity heatmap illustrates word clouds that the from 5134 GO terms in down-regulated oocyte biological function that have been clustered and annotated with word clouds. (C) The number of BP ontologies in each cluster (Top 15). (D) The PPI network shown the down-regulated proteins. (E) The top 30 hub genes within the PPI network were further abstracted. (F) KOG categories of differentially abundant proteins in D417N mutation oocytes.

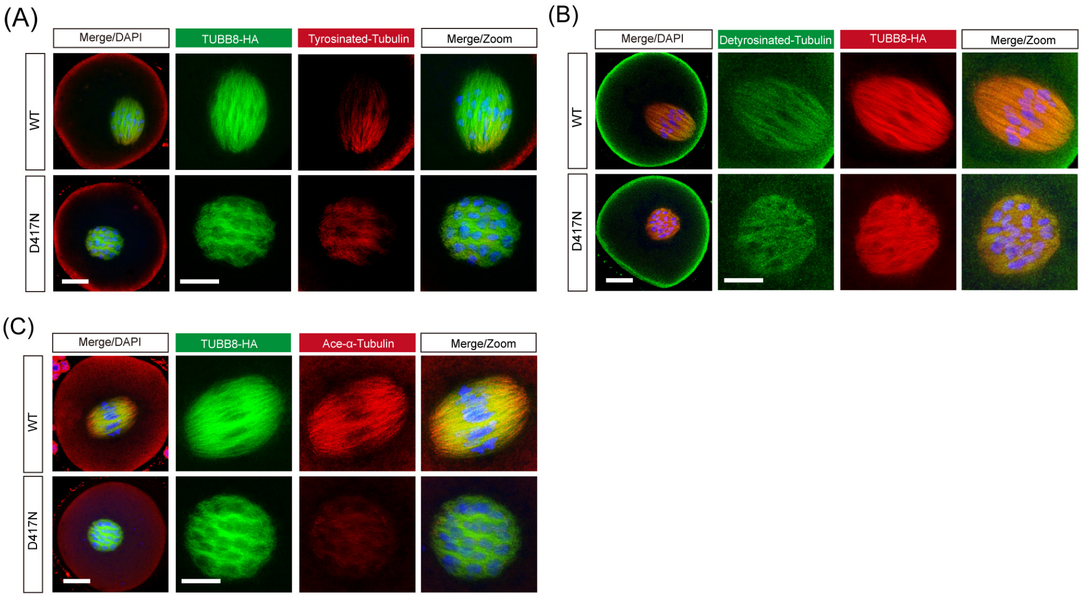

Figure S9. Staining of tubulin post-translational modifications on the meiosis I spindle. (A and B) Oocytes were fixed 5h after GVBD and stained for the indicated modifications on tubulin. Images are sum intensity z projections; scale bars, 10 μm. (C) Representative confocal images of spindle morphology in groups of oocytes from WT and D417N mutation. The metaphase I oocytes were immunostained with DAPI to visualize chromosomes (blue), TUBB8-HA to visualize microtubule (green), and Ace-α-Tubulin (red) (Sigma, T7451). Images are sum intensity z projections; scale bars, 10 μm.

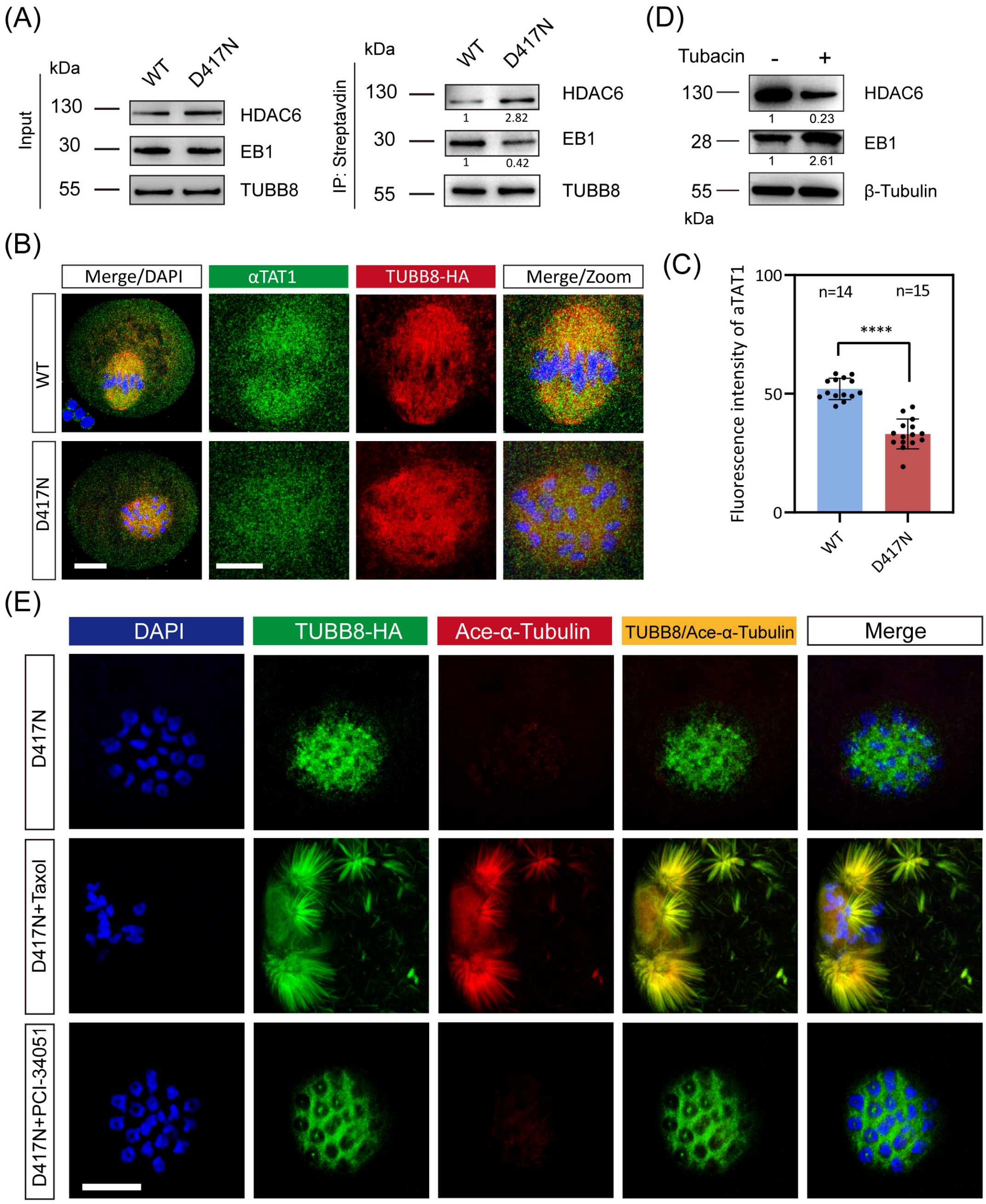

Figure S10. Tubacin treatment and non-HDAC6 inhibitors treatment for D417N mutation oocytes. (A) Co-immunoprecipitation assay was performed to determine the interactions between TUBB8 and HDAC6 or EB1 by D417N mutation. The blots of protein precipitants were probed with EB1, HDAC6, and streptavidin (TUBB8) antibodies respectively. (B) Representative immunofluorescence images of mouse metaphase I oocytes. Green, αTAT1; red, microtubule (TUBB8); blue, DNA (DAPI). Scale bar, 10 μm. (C) Quantitative analysis demonstrates the mean fluorescence intensity of αTAT1 (Unpaired Student's t test, two-tailed, **** $P<0.0001$). Numbers

indicate the individual oocytes quantified. (D) Inhibition of HDAC6 increases expression of EB1. Western blot of HeLa cells treated with DMSO (-) or Tubacin (+). β-Tubulin was probed as a loading control. (E) Representative confocal images of spindle morphology in groups of D417N (DMSO), D417N + 10 μM Taxol, and D417N + 10 μM PCI-34051. The metaphase I oocytes were immunostained with DAPI to visualize chromosomes (blue), TUBB8-HA to visualize microtubule (green), and ace-α-Tubulin (red). Scale bars, 10 μm.

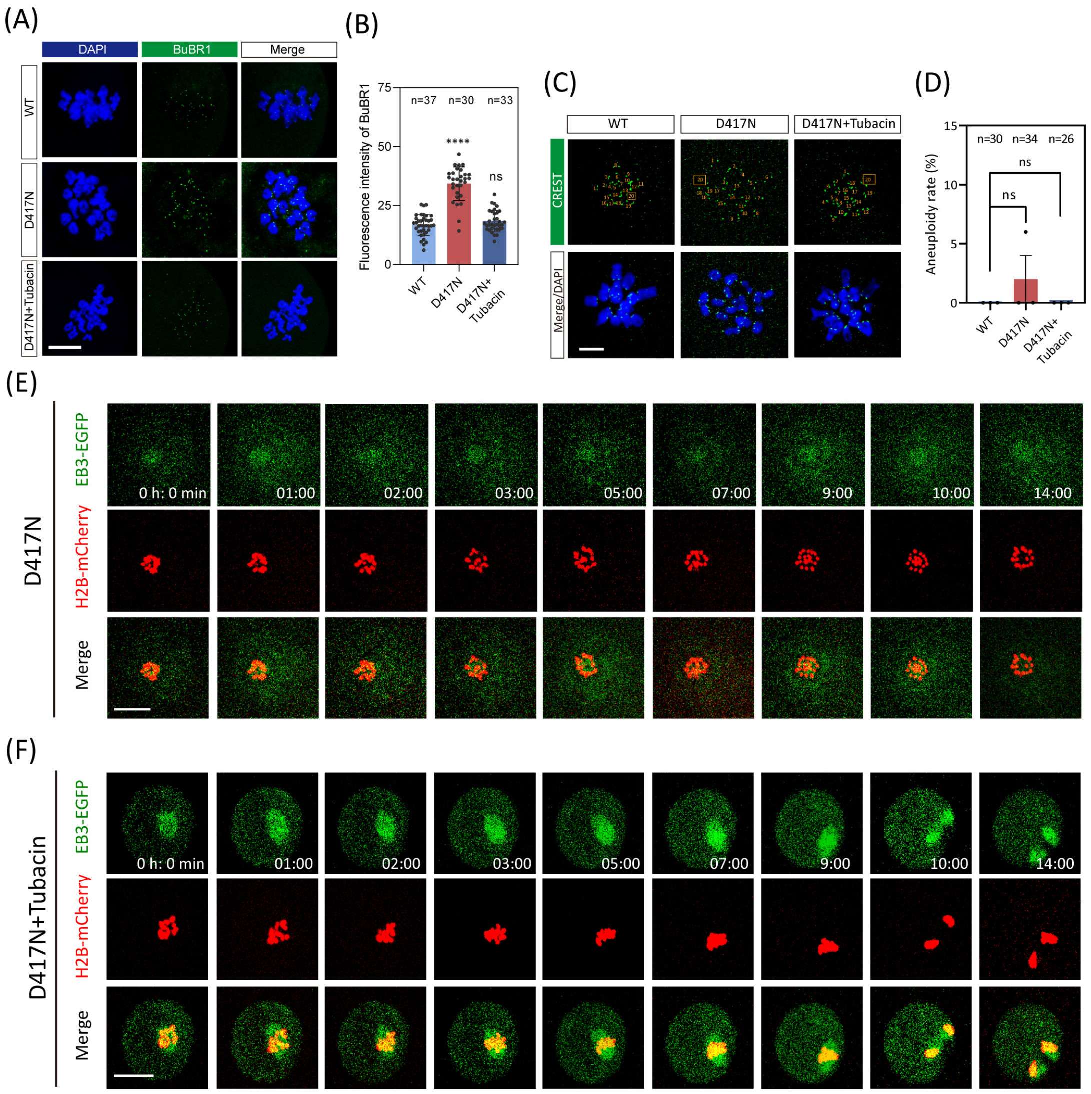

Figure S11. Tubacin could rescue D417N-mutant oocytes. (A) Representative immunofluorescence images of mouse metaphase I oocytes. Green, BuBR1; DNA, DAPI. Scale bar, 10 μm. (B) Quantitative analysis of mean BuBR1 fluorescence intensity for WT and D417N oocytes. Data were presented as the mean ± SEM. Numbers indicate the individual oocytes quantified. **** $P < 0.0001$ by One-way ANOVA with multiple comparisons test; ns, no significance. (C) Oocyte were treated with monastrol (100 μM) at 16 h after culture, and then oocytes were fixed and immunostained for centromere and DNA. Chromosome numbers are indicated in the images. Scale bar, 5 μm. Green, CREST; blue, DNA (DAPI). Scale bar, 10 μm. (D) Quantitative analysis of the aneuploidy rates. Data were presented as the mean ± SEM (One-way ANOVA with Šidák correction for multiple comparisons, ns= no significance). (E-F) Time-lapse imaging of a maturing oocyte expressing EB3-EGFP

(microtubule plus ends, green) and H2B-mCherry (chromosomes, red) in D417N (E) and D417N+Tubacin (F) oocytes. The scale bar is 25 μm. Time hh: mm relative to GVBD.

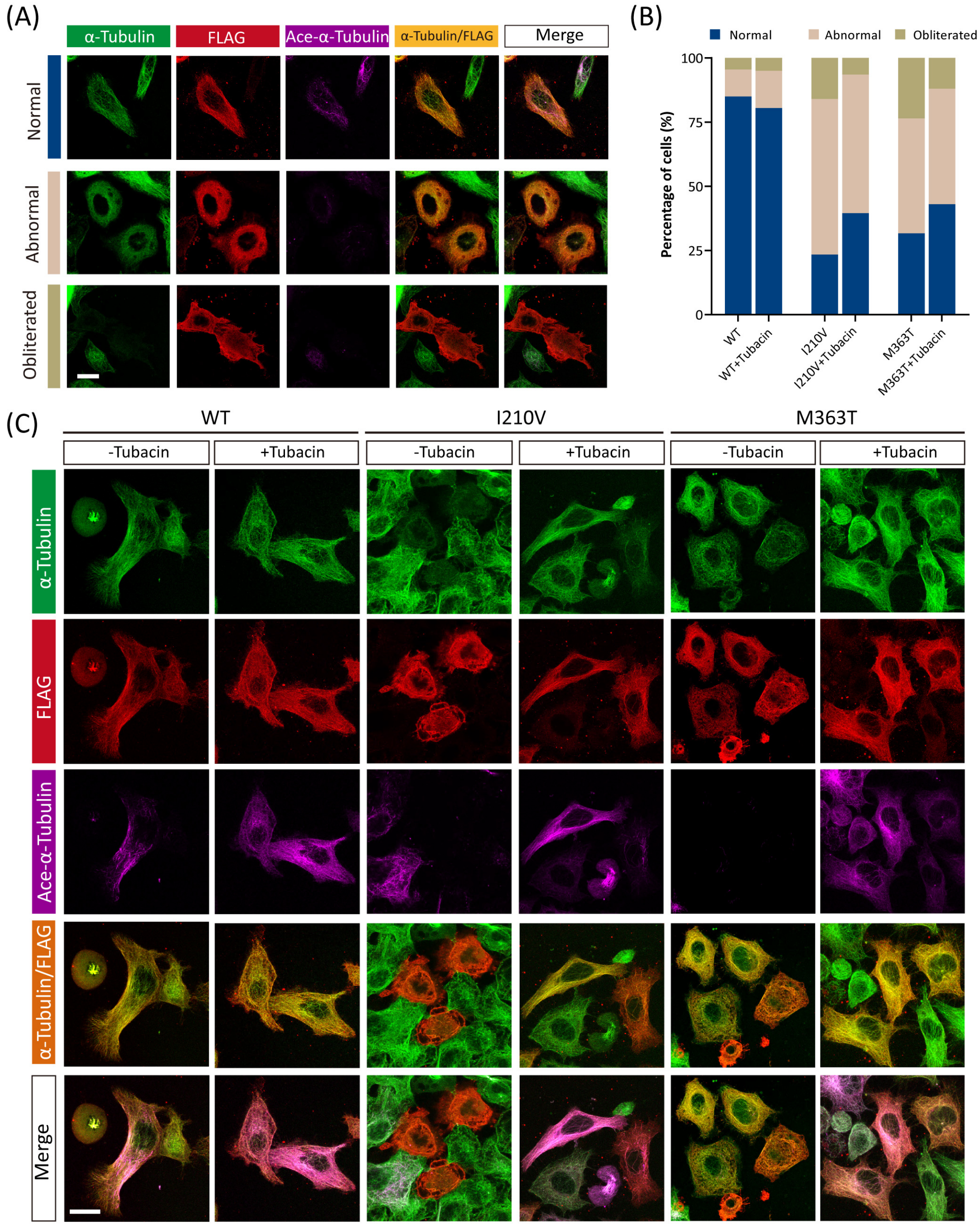

Figure S12. Microtubule morphology and phenotypes of TUBB8 WT and mutant in HeLa cells. (A) HeLa cells were transfected with constructs engineered to express TUBB8 (WT and mutant) fused with C-terminally Flag-tagged. The cells were immunostained with FLAG (to detect transgene, red), α-tubulin (to detect the endogenous microtubule network, green), and ace-α-tubulin (to detect the acetylated microtubule, magenta). The microtubule phenotypes (classified as normal, abnormal, or obliterated) were characterized and scored as described previously. (B) Quantitative

analysis of the microtubule phenotypes shown in panel (A). Approximately 200 transfected cells expressing either wild-type or mutant TUBB8 were examined in each of the three independent experiments. (C) Representative immunofluorescence images of Hela cells transfected with Flag-tagged encoding WT or missense variants of TUBB8. Green, β-tubulin; red, FLAG (TUBB8); magenta, acetylated microtubule (ace-α-Tubulin); blue, DNA (DAPI). Scale bars, 10 μm.

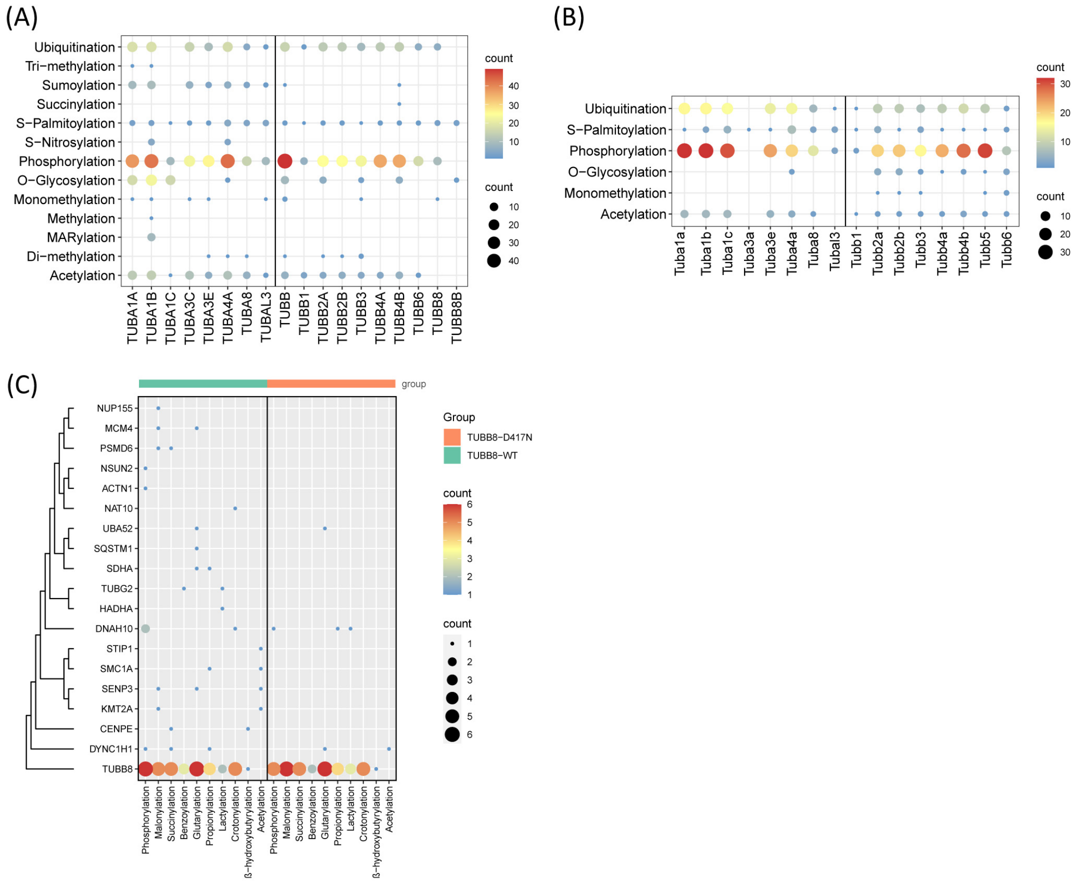

Figure S13. (A to B) Bubble maps illustrate the total number of various modifications in different isotypes of α-tubulin and β-tubulin in human (A) and mouse (B). (C) Proteomic post-translational modification analysis of TUBB8 and tts interacting proteins.

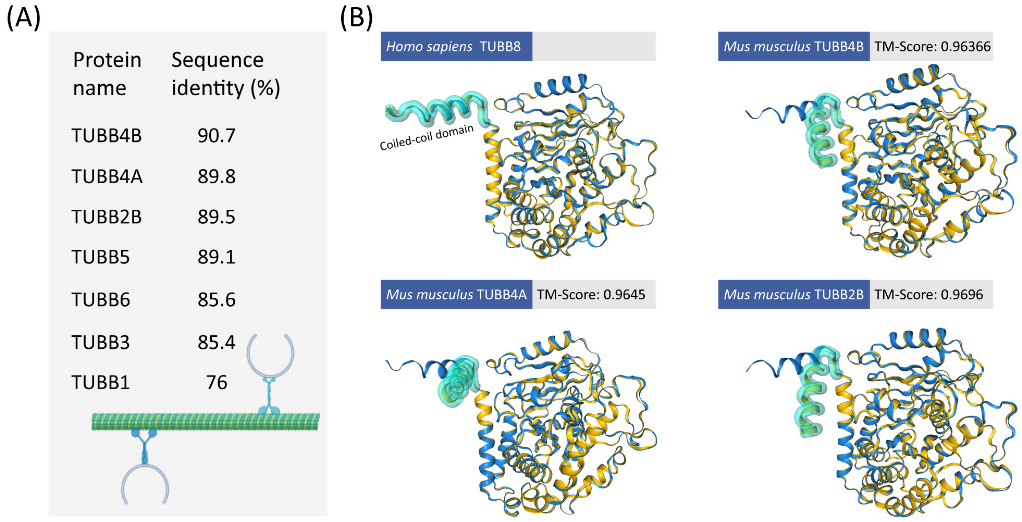

Figure S14. Predicted microtubule isotypes in mice resemble to human TUBB8 by Foldseek (Alphafold2-based). (A) A comparative analysis of tubulin sequence identity similar to TUBB8 in mice, (B) The protein structures of TUBB8 and its highly homologous protein in mice, specifically TUBB4B, TUBB2B, and TUBB2A, with notable differences highlighted for clarity. TM-Score, a metric for assessing the accuracy of protein structure prediction models.
